# Plasma Cell Myeloma: Biochemical Insights into Diagnosis, Treatment, and Smart Nanocarrier-Based Therapeutic Development

**DOI:** 10.3390/pharmaceutics17121570

**Published:** 2025-12-05

**Authors:** Lizeth Geraldine Muñoz, Sixta Palencia Luna, Andrés Felipe Chamorro

**Affiliations:** 1 Research Group of Electrochemistry and Environment (GIEMA), Faculty of Basic Sciences, Universidad Santiago de Cali, Cali 760035, Colombia; lizeth.munoz02@usc.edu.co; 2 Department of Biological Sciences and Chemical, Faculty of Sciences, Universidad San Sebastián, Lientur 1457, Concepción 4030000, Chile

**Keywords:** multiple myeloma, chemical diagnosis, monoclonal antibodies, proteasome inhibitors, nanomaterials, therapeutic resistance, CAR-T, immunotherapy

## Abstract

Plasma cell myeloma (PCM) is classified as a blood cancer and is characterized by the abnormal proliferation of plasma cells in the bone marrow and the excessive production of monoclonal immunoglobulins, which lead to permanent damage to vital organs. Although treatment strategies have improved with the development of proteasome inhibitors (PIs), immunomodulatory drugs (IMiDs), and monoclonal antibodies (mAbs), PCM remains an incurable disease due to its molecular heterogeneity and the development of drug resistance. In this review, we discuss the biochemical and molecular foundations underlying the diagnosis and treatment of PCM, emphasizing both traditional and advanced approaches. Classical methods such as serum protein electrophoresis (SPEP), immunofixation electrophoresis (IFE), and serum free light chain (sFLC) determination are highlighted alongside their integration with highly sensitive techniques like mass spectrometry (MS) and next-generation sequencing (NGS). Special attention is given to nanotechnology-based systems, including liposomes, polymeric nanoparticles (NPs), dendrimers, and hybrid nanocapsules, which enable controlled drug release, targeted delivery, and the minimization of systemic toxicity. Increasingly, nanomaterials are being shown to greatly enhance the biodistribution and pharmacokinetics of anticancer drugs, leading to improved therapeutic effects and escaping resistance mechanisms by employing multifunctional strategies that include dual drug co-encapsulation, pH-sensitive release and theranostic applications. Furthermore, the integration of nanotechnology with immunotherapy platforms represents a paradigm shift toward precision and personalized medicine for the treatment of PCM. Overall, this review views nanotechnology as an enabling technology to improve therapeutic effectiveness, minimize toxicity and open new avenues toward next-generation smart and personalized therapeutics for the treatment of PCM.

## 1. Introduction

Multiple myeloma, currently referred to as PCM, is a hematologic malignancy that affects the blood and bone marrow, the spongy tissue inside bones responsible for producing blood cells. In this disease, plasma cells, a type of white blood cell, undergo malignant transformation and proliferate uncontrollably. These abnormal cells secrete large amounts of defective antibodies, known as monoclonal proteins (M proteins), which accumulate in the blood and organs, disrupting their normal functions [1,2].

PCM accounts for approximately 1% of all solid tumor cases and about 10% of all hematologic malignancies [3]. According to estimates from the American Cancer Society (2025), around 36,110 new cases of PCM are expected to be diagnosed (20,030 in men and 16,080 in women), with an estimated 12,030 deaths. The average lifetime risk of developing this cancer is less than 1%, corresponding to 1 in 108 men and 1 in 133 women. Age is one of the main risk factors, with fewer than 1% of cases diagnosed in individuals under 35 years old and the majority occurring in those over 65, with a median age at diagnosis of 69 years [4]. In Europe, the prevalence of PCM ranges from 6 to 8 cases per 100,000 population, whereas in Latin America it ranges from 2 to 4 cases per 100,000 [3]. Before the clinical diagnosis of PCM, some patients experience an asymptomatic phase known as monoclonal gammopathy of undetermined significance (MGUS), which is characterized by the presence of small amounts of M protein in the blood without evidence of organ damage. Since only a small proportion of MGUS cases progress to active myeloma each year (approximately 1%), early detection is essential to enable timely disease management [5].

The diagnosis of PCM involves a combination of biochemical tests and imaging studies. M protein can be detected in 80–90% of cases using (SPEP), while more specific methods such as IFE and MS can achieve sensitivities of up to 95% [6,7]. Additionally, over the past decades, the treatment of PCM has evolved dramatically, where bone and bone marrow lesions can be detected by computed tomography (CT) and magnetic resonance imaging (MRI) in approximately seven out of ten patients [8]. Classical treatment methods, such as chemotherapy, corticosteroids, and hematopoietic stem cell transplantation (HSCT, bone marrow transplantation), are now complemented by more specific agents, including PIs (e.g., bortezomib) and mAbs (e.g., daratumumab), which have increased the median survival from a few years to approximately eight years [8,9,10]. Nevertheless, about 60% of patients eventually develop long-term resistance to therapy.

To address this challenge, nanomedicine has emerged as a powerful tool. The use of Nps capable of delivering drugs specifically to malignant cells has been shown to increase therapeutic efficacy and reduce side effects by up to 40% [11]. Furthermore, crucial biochemical processes involved in cancer development, such as intracellular signaling pathways including nuclear factor kappa-light-chain-enhancer of activated B cells (NF-κB), phosphatidylinositol 3-kinase/protein kinase B (PI3K/Akt), and Janus kinase/signal transducer and activator of transcription (JAK/STAT), have been recognized as major regulators of cell proliferation and survival [12]. Targeted therapies directed against these pathways, along with novel immunological strategies such as chimeric antigen receptor T-cell (CAR-T) therapy, based on genetically modified T lymphocytes that bind to tumor antigens, have achieved success rates of up to 80%. However, severe adverse events associated with allogeneic HSCT occur in approximately 20% of patients [13,14]. On the other hand, there remains a gap in the literature that comprehensively integrates the biochemical, diagnostic, and therapeutic aspects of PCM. Therefore, this review focuses on the biochemical implications of the disease, emphasizing biomedical chemistry and nanotechnology approaches as the foundation for developing more precise diagnostic tools and more effective and safer treatments.

## 2. Pathophysiology and Molecular Basis of PCM

**Figure 1:**
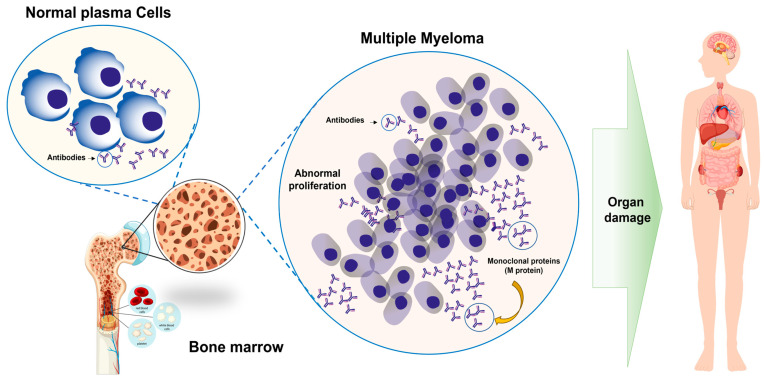
Normal plasma cell versus deregulated proliferation in PCM. Plasma cells, derived from B lymphocytes, white blood cells responsible for producing antibodies, undergo malignant transformation, leading to uncontrolled proliferation within the bone marrow. This process disrupts normal blood cell production and leads to the progressive damage of vital organs, including bones and kidneys, due to an overaccumulation of M protein.

Among the main pathogenic processes are (i) the activation of intracellular signaling pathways [15]; (ii) genomic and epigenetic instability, resulting from point mutations, chromosomal deletions, and chemical modifications of deoxyribonucleic acid (DNA), alters gene expression and generates clonal heterogeneity [6,8]; and (iii) interaction with the bone marrow microenvironment [7]. The overproduction of M protein by tumor cells also leads to progressive kidney damage and immunosuppression, as illustrated in Figure 1.

PCM typically develops from MGUS, which occurs in a proportion of individuals over 50 years of age, of whom only a fraction progress to active myeloma [6]. This switch is thought to be related to the acquisition of novel genetic mutations and changes in the bone marrow microenvironment that promote the survival and clonal expansion of malignant cells [16,17]. Diagnosis is based on the CRAB criteria (hypercalcemia, renal impairment, anemia, and bone lesions) supplemented by the quantification of plasma cells in the bone marrow [8,18]. Deciphering the above mechanisms is crucial to pave the way for more tailored diagnostics and therapeutic interventions to ultimately enhance patient survival and quality of life [19]. The following Section 2.1, Section 2.2 and Section 2.3 describe the molecular and biochemical aspects of myeloma, the genetic and epigenetic alterations, and the principal serum biomarkers involved in its pathophysiology.

### 2.1. Malignant Transformation and Genetic Alterations in PCM

PCM is a blood cancer with uncontrollably growing abnormal plasma cells in the bone marrow, the spongy tissue inside bones responsible for producing red blood cells, white blood cells, and platelets, which in turn mediate oxygen transport, immune defense, and blood clotting, respectively [19]. These plasma cells arise from B lymphocytes, a type of white blood cell that participates in the adaptive immune response and that under normal circumstances secretes functional antibodies capable of neutralizing organisms. In PCM, plasma cells undergo genetic and biochemical alterations [20,21].

From a genetic perspective, malignant plasma cells exhibit multiple mutations, chromosomal translocations, and deletions that affect key genes involved in cell cycle regulation and DNA repair. Among the most frequent translocations are those between chromosomes 11 and 14 [t(11;14)], chromosomes 4 and 14 [t(4;14)], and chromosomes 14 and 16 [t(14;16)]. In addition, deletions of the long arm of chromosome 13 (13q) and mutations in genes such as KRAS (Kirsten rat sarcoma viral oncogene homolog), NRAS (neuroblastoma rat sarcoma viral oncogene homolog), FGFR3 (fibroblast growth factor receptor 3), and MYC (v-myc avian myelocytomatosis viral oncogene homolog) are associated with more aggressive disease forms and poorer responses to conventional treatments [22,23]. These alterations activate oncogenes that promote cell proliferation and tumor formation while inactivating tumor suppressor genes, whose role is to prevent uncontrolled cell growth. Collectively, these molecular changes drive the expansion of malignant clones and their capacity to infiltrate organs such as the bone and kidney [24].

For instance, hypermethylation of the promoter regions of tumor suppressor genes such as cyclin dependent kinase inhibitor 2A (p16INK4a) and RAS association domain family member 1, isoform A (RASSF1A) results in the silencing of these genes, while changes in histone acetylation affect DNA accessibility and regulate diverse cellular processes [24]. Moreover, microRNAs (miRNAs), small non-coding RNAs transcribed at the post-transcriptional level that control gene expression, are also associated with cell viability. Specifically, the upregulation of miR-21 and downregulation of miR-15a/16 suppress proapoptotic gene expression and contribute to the survival of the malignant clone [25,26]. These mutations and epigenetic changes affect intracellular signaling pathways such as mitogen activated protein kinase (MAPK) and PI3K/AKT, which regulate cell growth, differentiation, and survival. When aberrantly activated, these pathways enable malignant plasma cells to evade apoptosis, persist in the bone marrow, and form clones that produce large amounts of M protein and sFLCs (serum free light chains) of immunoglobulins, thereby altering plasma composition and generating systemic complications [26,27].

These signaling cascades are central to the survival of malignant plasma cells. Activation of the NF-κB pathway, commonly driven by cytokines such as IL-6 and TNF-α within the bone marrow microenvironment, induces the transcription of pro-survival genes including BCL-XL, cIAP1/2, and MCL-1, thereby suppressing apoptosis and enhancing inflammatory signaling [12,21]. Similarly, aberrant activation of the PI3K/Akt pathway promotes tumor progression by inhibiting key apoptotic mediators such as BAD and caspase-9, while simultaneously upregulating antiapoptotic proteins from the BCL-2 family [15,26]. Akt activation also increases glucose metabolism and protein synthesis, sustaining the high metabolic demands of myeloma cells [24]. In parallel, the JAK/STAT axis particularly STAT3mis stimulated by IL-6 and other growth factors and acts as a transcriptional driver of MCL-1, the dominant antiapoptotic protein in plasma cell myeloma and a critical mediator of drug resistance [12,21]. Collectively, these interconnected pathways converge on the overexpression of BCL-2 and MCL-1, enabling malignant plasma cells to evade programmed cell death, resist chemotherapeutic stress, and maintain clonal expansion within the bone marrow niche [12,15]. The bone marrow microenvironment, composed of stromal cells, osteoblasts, osteoclasts, cytokines, and growth factors such as interleukin-6 (IL-6) and vascular endothelial growth factor (VEGF), establishes a communication network with malignant cells that reinforces their proliferation, promotes angiogenesis, and contributes to therapeutic resistance, while also favoring bone resorption and the release of calcium into the bloodstream [21,27].

**Figure 2:**
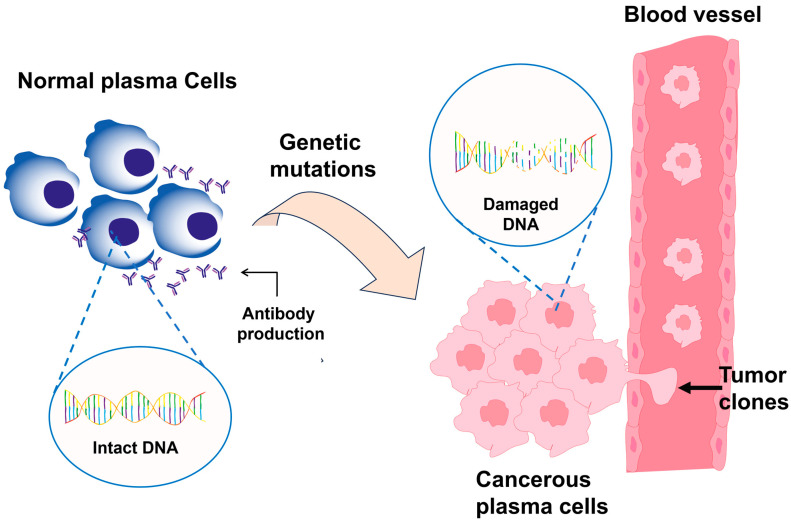
Comparison between a normal plasma cell and a malignant plasma cell in PCM. The healthy cell maintains intact DNA and produces functional antibodies, whereas the malignant cell presents genetic mutations and epigenetic alterations that activate uncontrolled growth pathways, promoting proliferation, resistance to apoptosis, and excessive production of M protein.

As illustrated in Figure 2, the comparison between a normal plasma cell and a malignant plasma cell demonstrates how the intact DNA structure and functional antibody production of the healthy cell are replaced by genetic mutations, epigenetic alterations, and uncontrolled activation of growth pathways in the cancerous cell, thereby facilitating continuous proliferation and resistance to apoptosis. Collectively, these figures provide a more detailed view of molecular events that lead to the development of PCM and its relevance in clinical practice [28]. Detection of these alterations is important for the development of specific treatments, such as histone deacetylase inhibitors, gene therapies, or strategies using nanotechnology to restore the genetic regulation, inhibit the tumor growth, and reduce adverse effects [22,28]. According to the above, the transformation of plasma cells into malignant cells, the accumulation of M protein, and the presence of genetic mutations and epigenetic alterations constitute the molecular pillars that explain the evolution of PCM, its therapeutic resistance, and its capacity to affect vital organs. The ability to understand these mechanisms enables a more personalized disease management, such as correct diagnosis, specific therapy, and surveillance of disease-specific biomarkers.

### 2.2. Proteins and Serum Biomarkers Involved in the Pathophysiology of PCM

Proteins and serum biomarkers are crucial in the pathophysiology of PCM, as they indicate the tumor load and the metabolic as well as systemic activity of the disease. The study of these biomarkers has enabled the establishment of direct correlations between the production of abnormal monoclonal immunoglobulins, alterations in the bone marrow microenvironment, and the characteristic clinical manifestations, making them indispensable tools for diagnosis, staging, and therapeutic monitoring [1,6,22]. The M protein is the major biochemical marker of PCM. Its quantification through SPEP and IFE enables the assessment of tumor clone magnitude and the monitoring of therapeutic response. In non-secretory myeloma, if the M protein is not detected, sFLC analysis is essential. These κ (kappa) or λ (lambda) light chains circulate freely in plasma, and their κ:λ ratio (normal range: 0.26–1.65) is significantly altered in the presence of monoclonal proliferation. A pathological κ:λ ratio (<0.01 or >100) serves as a sensitive indicator of tumor activity, even in the absence of detectable M protein [29,30].

Another important biomarker is β2-microglobulin (β2M), a protein associated with the class I major histocompatibility complex (MHC-I), found on the membrane of nearly all nucleated cells in the body, except red blood cells. Its serum concentration is influenced by both tumor burden and renal function, making it a powerful prognostic indicator [22]. Serum β2M levels greater than 5.5 mg/L correlate with poorer overall survival (OS), defined as the time from diagnosis or treatment initiation to death from any cause, and with higher resistance to therapy. Consequently, β2M was incorporated, along with serum albumin, into the International Staging System (ISS) for PCM, enabling stratification of patients according to clinical risk and tumor burden [31]. Lactate dehydrogenase (LDH), a cytosolic enzyme that catalyzes the reversible conversion of lactate to pyruvate in glycolytic metabolism, serves as an indirect marker of tumor proliferation and tissue destruction. In PCM, serum LDH levels greater than 270 U/L are associated with a more aggressive disease phenotype and significantly shorter progression-free survival (PFS), which is the time from diagnosis or treatment initiation until disease progression or patient death, thus representing a marker of high biological risk [1].

Bone involvement, a pathophysiological hallmark of PCM, results from the predominance of osteoclastic over osteoblastic activity. Osteocalcin, a non-collagenous protein secreted by osteoblasts that undergoes γ-carboxylation, is used to assess active bone formation; a decrease in its serum concentration is associated with osteoblastic inhibition [1,8]. In contrast, bone resorption and osteoblast proliferation are positively reflected by the levels of alkaline phosphatase, an enzyme expressed by osteoblasts. Elevated osteoblast marker bone alkaline phosphatase (BAP) and osteoclast marker tartrate-resistant acid phosphatase (TRAP) levels are indicative of high bone turnover. This biochemical imbalance manifests clinically as bone pain, pathological fractures, and lytic lesions, radiological hallmarks of myeloma [8].

On the molecular side, multiple genetic and cytokine biomarkers have been discovered that offer supplementary prognostic information. Mutations in genes such as tumor protein 53 (TP53), KRAS, and MYC are associated with increased disease aggressiveness, therapeutic resistance, and early relapse [32]. These alterations can be detected using quantitative real-time PCR (qPCR) or NGS, techniques that enable high-sensitivity evaluation of the genomic profile and mutational burden [32,33,34]. Table 1 summarizes the quantitative values, determination methods, clinical manifestations, and diagnostic and prognostic applications of the most relevant biochemical and molecular biomarkers. As discussed, these parameters constitute the foundation for the biological characterization of PCM, reflecting both tumor activity and the extent of systemic damage. The combined assessment of protein, enzymatic, and genetic markers enables the establishment of an integrated disease profile that is valuable for risk stratification and therapeutic response monitoring [1,6,22].

Among the classical biomarkers, M protein remains the most representative indicator of tumor burden. Its quantification by SPEP and IFE allows the detection of concentrations above 0.5 g/L with high reproducibility. However, high-resolution techniques such as MS and tandem MS (MS/MS) have expanded analytical sensitivity by up to 1000-fold, enabling the identification of minimal residual disease (MRD, defined as the persistence of small amounts of malignant cells following therapy), the small population of cancer cells that persists in the body after treatment, even when the patient is in complete clinical remission, long before clinical or radiological signs of relapse appear [35,38]. This enhanced detection capability has been essential for the early prediction of relapses and timely therapeutic modulation, although the lack of methodological standardization currently limits its widespread implementation. The detection of sFLC and the κ/λ ratio serves as a valuable complement to M protein identification, particularly in non-secretory myelomas where M protein cannot be quantified. Automated immunochemical assays (Freelite^®^ or N Latex FLC) provide diagnostic sensitivities of 90–95% and enable the detection of subtle alterations in the κ:λ ratio (reference range: 0.26–1.65). Patients with values outside this range, particularly those with sFLCR ≥47 or ≤0.01, exhibit a higher risk of disease progression and reduced OS [22,39]. The addition of this marker to SPEP and MS increases the separation of active from indolent and early relapsing myelomas, supporting the prognostic import of this beaker.

β2M is a well-established prognostic factor incorporated into the ISS. Its increase is due to both tumor load and renal insufficiency caused by the overproduction of monoclonal immunoglobulin. Serum levels > 5.5 mg/L are associated with reduced PFS and increased treatment resistance, making β2M one of the most powerful parameters for clinical stratification of PCM [22,31]. In contrast to M protein, β2M is not dependent on tumor secretion, making it a useful marker in non-secretory or incompletely remitted disease as well. Metabolically, LDH is linked to tumor proliferation rate and cellular destruction; levels ≥ 271 U/L indicate high-risk disease and correlate with a PFS of less than 12 months [1]. It is not specific because it can also rise in inflammatory or infectious states; however, when combined with β2M and serum albumin, LDH does have prognostic value.

In conclusion, the data from Table 1 suggest that the proteomic and molecular profile of PCM represents a multimodal diagnostic approach in which classical biochemical markers, indicators of tissue damage, and genomic biomarkers are integrated to provide a comprehensive view of the disease. The interplay of the M protein levels, sFLC ratio, β_2_M, LDH, and somatic mutations allows tailored monitoring and treatment, enabling customized treatment and follow-up protocols. Thus, the synergy of these biomarkers not only improves diagnostic accuracy but also paves the way for precision medicine in PCM management and surveillance [37,39].

While the majority of the biomarkers discussed above are clinically validated and are routinely employed in the aid of diagnosis, risk stratification, and therapy monitoring, a handful of promising candidates have been described in recent years which are not yet sufficiently validated for clinical application. Among them, circulating extracellular vesicles (EVs) enriched in CD138^+^ plasma cell markers, serum galectin-9, and soluble BCMA (sBCMA) have shown potential for detecting early relapse and capturing dynamic tumor changes; however, significant interassay variability and the absence of standardized thresholds limit their widespread use [22,34]. Comparably, metabolomic profiles including changes in acylcarnitines, lysophosphatidylcholines and certain amino acid derivatives have shown correlations with disease activity and response to treatment in exploratory series, but cross-platform reproducibility is poor [25]. Additional candidates, including circulating tumor DNA (ctDNA) burden and immune checkpoint molecules such as soluble PD-L1, have yielded promising preliminary data but require larger prospective studies to establish sensitivity, specificity, and prognostic value compared with established markers [32]. As these analytes are further studied, implementation in the clinic will require harmonization of methodology, validation in multiple centers, and demonstration of added value relative to contemporary standards of diagnosis [39].

### 2.3. Bone Marrow Microenvironment and Factors Associated with Tumor Progression

The bone marrow microenvironment is a multifaceted biological system that is critical for the proliferation, survival and advancement of PCM. It includes a variety of cell populations, such as stromal, bone, and endothelial cells, and an extracellular matrix (ECM), along with a web of soluble factors like cytokines, chemokines, and growth factors, which promote cell proliferation, differentiation, survival, and migration. These components together control important processes like cell growth and division, differentiation, and cell death (apoptosis). If this balance is upset a microenvironment is created that promotes survival of tumor clones, resistance to therapy, and invasion into adjacent tissues [36,40]. Interactions between tumor cells and stromal cells are one of the key pillars of this pathological environment. Studies have shown that under hypoxic conditions (low oxygen concentration), communication between these two cell populations can increase the survival of malignant plasma cells by up to 3.5-fold [40]. Similarly, drug resistance is increased by 48% when plasma cells are co-cultured with bone marrow fibroblasts, indicating that bone marrow microenvironment-derived signals provide protection against antineoplastic agents [36,40].

Among the most relevant soluble mediators are cytokines and chemokines that regulate cell proliferation and migration. Background IL-6, a proinflammatory cytokine secreted by stromal cells, is a direct growth and survival factor for malignant plasma cells. An increase in proliferation of plasma cells by about 65% has been observed in vitro at serum IL-6 levels above 10 pg/mL [41]. The C-X-C motif chemokine ligand 12 (CXCL12), also known as stromal-derived factor 1 (SDF-1), acts as an anchoring molecule that retains malignant plasma cells within the bone marrow niche [41]. CXCL12 levels >100 ng/mL have been demonstrated to promote tumor cell retention in the bone marrow microenvironment which contributes to drug resistance and immune clearance resistance. Both molecules synergize to generate a protective niche that fosters tumor persistence [42].

The ECM is made up of proteins, such as collagen, fibronectin, and proteoglycans, and is a three-dimensional structure, which is found to be important in the development of PCM. Tumor cells adhering to ECM via β1 integrins and N-cadherins have been reported to enhance apoptotic resistance in around 40% of cases [43]. Concurrently, elevated expression of matrix metalloproteinases (MMPs), particularly MMP-2 and MMP-9, promotes ECM degradation and enhances tumor cell invasiveness by about 55% [43]. Altogether, these results suggest that matrix adhesion and degradation are intertwined events that contribute to tumor cell invasion and proliferation [43]. Angiogenesis is another key process in PCM and refers to the formation of new blood vessels from pre-existing ones. This effect is mainly mediated by VEGF, a protein stimulating the formation of a capillary network in the environment of the bone marrow. An increase of approximately 42% in bone marrow microvascular density has been observed in association with VEGF levels exceeding 300 pg/mL [44], providing enhanced oxygenation and nutrient supply to tumor cells. Nevertheless, this hypervascularization is not consistently proportional to tumor load and could represent an adaptive response to hypoxia [44].

**Figure 3:**
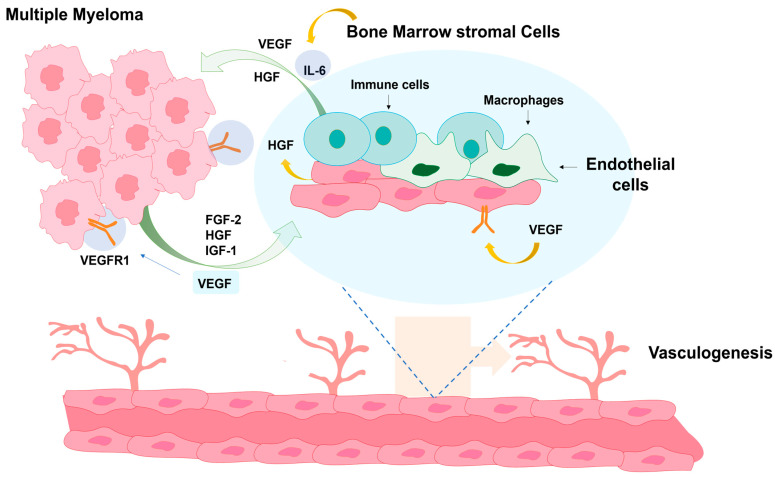
Cellular interactions within the bone marrow microenvironment in PCM. The illustration depicts communication between myeloma cells, stromal cells, endothelial cells, macrophages, and immune cells through signaling molecules such as VEGF, HGF, FGF-2, IGF-1, and IL-6, which collectively promote tumor growth, survival, and vasculogenesis.

When analyzed from an immunological perspective, the bone marrow cavity is considered immunosuppressive. Studies have reported a 60% expansion of regulatory T cells (Tregs), which suppress the antitumor immune response, along with a substantial proliferation of tumor-associated macrophages (TAMs) that secrete anti-inflammatory cytokines facilitating neoplastic progression. In parallel, a 35% reduction in cytotoxic T lymphocyte activity has been observed, diminishing the clearance of cancer cells and contributing to the perpetuation of a tumor-tolerant microenvironment. As shown in Figure 3, the bone marrow microenvironment in PCM functions as a dynamic network of interactions among myeloma cells, bone marrow stromal cells, endothelial cells, macrophages, and immune cells. Myeloma cells secrete angiogenic factors such as VEGF, hepatocyte growth factor (HGF), fibroblast growth factor-2 (FGF-2), and insulin like growth factor-1 (IGF-1), thereby enhancing cell proliferation and survival. These bidirectional signals trigger endothelial activation and vasculogenesis, leading to the generation of new blood vessels that support tumor growth and metastasis.

## 3. Diagnostic Methods in PCM

The diagnosis of PCM requires a series of specialized biochemical, molecular and radiological investigations that are intended to confirm diagnosis, quantify disease load and guide therapeutic decisions. Given the biological complexity of PCM, the currently applied diagnostic framework adopts a multimodal approach that detects not only structural alterations in the bone marrow but also the molecular and metabolic changes associated with the proliferation of malignant plasma cells [45,46]. To obtain a correct description of disease, diagnostic examinations may be grouped into five main synergistic types:

(i) Conventional biochemical methods, including SPEP and IFE, which are traditionally employed owing to their low cost and wide availability, as well as their ability to identify monoclonal proteins or paraproteins in serum and urine;
(ii) Serum and urinary biomarkers (e.g., β2M, albumin, sFLC), which are essential for prognostic stratification as indicators of tumor burden, patient functional status, and therapeutic response [31,47];
(iii) Advanced spectrometric and analytical chemistry techniques, such as MS and NGS, which provide sensitivities up to a thousand times higher than conventional methods, enabling accurate quantification of M protein or clonotypic peptides, unique peptides derived from the variable (clonal) regions of immunoglobulins produced by malignant plasma cells in PCM, as well as the detection of MRD [35,48];
(iv) Molecular imaging techniques, including MRI and positron emission tomography/computed tomography (PET/CT), which offer a biochemical complement by providing both anatomical and functional evaluation of bone and extramedullary disease, allowing for therapy monitoring and detection of progression foci;
(v) Comparative assessment of diagnostic systems, aimed at evaluating the sensitivity, specificity, and clinical feasibility of each method in order to establish optimized protocols and improve early-stage detection [49].

**Figure 4:**
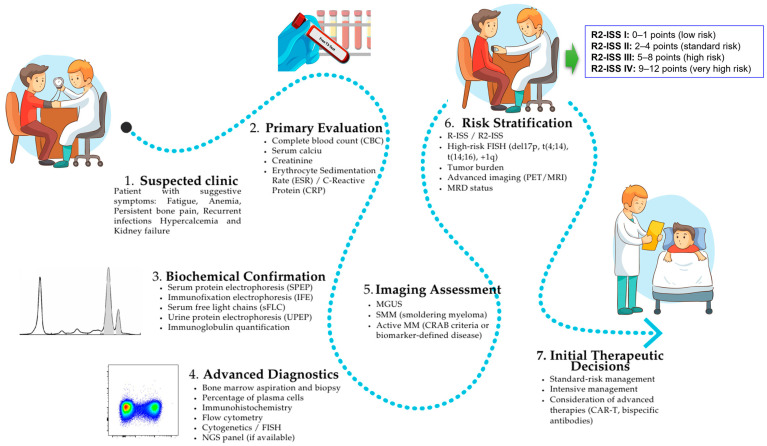
Flowchart illustrating the stepwise diagnostic workflow for PCM in Family and Clinical Medicine (FCM). The diagram integrates initial clinical assessment, first-line laboratory tests, specific biochemical analyses, advanced molecular diagnostics, and imaging modalities, culminating in final diagnostic classification and risk stratification according to IMWG criteria. This review highlights interaction of multimodal investigations in promoting precise diagnosis, staging and early treatment decisions.

To facilitate a unified understanding of how these diagnostic modalities are applied in routine clinical practice, a comprehensive overview of the stepwise diagnostic workflow is presented in Figure 4. This schematic summarizes the sequential integration of initial clinical assessment, biochemical testing, advanced molecular analyses, and imaging modalities, culminating in the final diagnostic classification and risk stratification recommended by current IMWG guidelines.

These combined strategies facilitate the establishment of a hybrid diagnostic scheme that leverages the accessibility and reliability of biochemical assays alongside the precision of molecular and imaging modalities, resulting in a deeper characterization of the disease. This multidimensional approach provides better sensitivity for early detection and prognostic stratification, and potentially also for therapeutic decisions in the context of personalized medicine. The following Section 3.1, Section 3.2, Section 3.3, Section 3.4 and Section 3.5 discuss the key techniques relevant to the diagnosis of PCM.

### 3.1. SPEP, IFE, and sFLC Determination

Biochemical diagnosis continues to be a cornerstone for suspicion and follow-up of PCM, since it allows the detection of M protein and other atypical products of the malignant plasma cells. Among the classical methods, the most commonly employed are SPEP, IFE, and sFLC analyses, which represent the cornerstone of biochemical diagnosis in PCM. SPEP is a technique that separates serum proteins into distinct fractions (albumin, α1-, α2-, β-, and γ-globulins) by applying an electric field [50]. The sensitivity of this method depends directly on the concentration of M protein, allowing the detection of levels equal to or greater than 0.5 g/dL. However, this technique presents limitations in non-secretory myelomas, where tumor cells fail to produce detectable amounts of immunoglobulin, and in the early stages of the disease, with an estimated false-negative rate of 20–30% [50,51].

To increase diagnostic specificity, IFE is employed, combining electrophoresis with the use of specific antibodies directed against the various heavy (IgG, IgA, IgM, etc.) and light chains (κ and λ) of immunoglobulins. This technique enables confirmation of the M protein identity and differentiation among immunoglobulin subtypes, increasing diagnostic specificity to over 95% [51]. Nevertheless, its sensitivity is limited for detecting MRD, as it identifies only 50–60% of cases in complete remission. Therefore, IFE is primarily used for initial diagnostic confirmation rather than for long-term patient monitoring [52].

The analysis of sFLC represents a major advancement in the diagnosis of PCM. These κ and λ chains are immunoglobulin fragments normally produced in small quantities but secreted in excess in myeloma due to the abnormal activity of neoplastic plasma cells. Quantitative sFLC immunoassays, such as Freelite^®^, N Latex^®^, and Sebia^®^, enable measurement of both light chains and calculation of the κ/λ ratio (sFLCR), whose alteration serves as an early indicator of tumor clonality [51,52]. These assays reach 90–95% sensitivity in detecting non-secretory and early-stage myelomas, thus becoming an excellent add-on to conventional electrophoretic methods [53]. Nonetheless, discrepancies between commercial platforms have been reported, with differences of up to 15% in measured values [53]. Such discrepancies indicate that there should be standardized procedures for interlaboratory analyses, and type-specific correction factors for diagnostic cutoffs should be established in a way that is safe in clinical practice. Also, serum light chain levels may be impacted by other factors, including renal dysfunction and inflammatory states, highlighting the need for result interpretation in the context of the clinical picture.

Accordingly, the available data support the use of SPEP, IFE, and sFLC analysis as a robust primary screening strategy, compensating for the inherent limitations of each individual technique. The combination of the above methods allows for a better understanding of tumor biology and the basis for the development of more complex, multimodal diagnostic algorithms. However, their role is increasingly challenged by newer technologies, such as MS and NGS, which offer 100- to 1000-fold higher sensitivity, enabling the detection of subclinical recurrences and more precise quantification of MRD [51,52,53]. However, traditional methods have clinical value because they are cheap and can be obtained in any hospital laboratory. Consequently, the current diagnostic paradigm is evolving toward a complementary approach, in which conventional techniques continue to serve as the first-line detection tools, while novel molecular technologies provide high-sensitivity confirmation and longitudinal monitoring in PCM.

### 3.2. Biochemical Biomarker Tests in Blood and Urine

Tests for biochemical biomarkers in blood and urine are fundamental for the diagnosis and follow-up of PCM. Apart from their pathophysiological relevance (discussed in previous sections), we consider here the aspect of quantitative determination and diagnostic sensitivity, specificity and utility. The M protein is still the reference parameter to which the confirmation of the diagnosis of PCM is referred. Its detection by SPEP or IFE reaches sensitivities of 0.5–1.0 g/dL, allowing detection of most of the active myeloma cases [54,55]. In contrast, MS, which separates molecules based on their mass-to-charge ratio, can detect concentrations up to 1000 times lower than conventional methods, allowing relapse detection up to 12 months earlier. Nevertheless, the absence of standardization and the high price of devices nowadays hamper its application in daily medical practice [56,57].

sFLC analysis complements M protein detection for disease monitoring, particularly in non-secretory myeloma. This assay measures free κ and λ light chains in plasma and the sFLC ratio (sFLCR) is calculated; a modified ratio is suggestive of monoclonal plasma cell proliferation. Immunoassays, such as ELISA or chemiluminescence, achieve sensitivities of 90–95%; however, interlaboratory variations of up to 15% have been reported, highlighting the need for standardized analytical procedures [52,58]. Bence Jones proteins, which are free immunoglobulin light chains (κ and λ) produced by malignant plasma cells and excreted in urine, remain classic indicators of tumor burden and renal injury. However, their assessment has largely been superseded by sFLC quantification, which is more convenient, less invasive, and diagnostically equivalent or superior [59]. Nevertheless, Bence Jones protein measurement may still be useful in evaluating suspected immunoglobulin-associated nephropathy, complementing renal workup.

Serum creatinine, microalbuminuria, and cystatin C remain the primary markers for monitoring PCM-induced kidney damage and guiding therapy adjustments based on glomerular filtration rate. Similarly, inflammatory markers such as C-reactive protein (CRP) and fibrinogen provide indirect information on systemic inflammation and tumor aggressiveness, although their prognostic value remains under debate [60,61]. Quantification of mRNA from genes such as IL-6 and VEGF, as well as the detection of somatic mutations by NGS, has demonstrated high diagnostic and prognostic value [62]. For instance, co-culture experiments revealed that VEGF stimulation of bone marrow stromal cells increased IL-6 secretion 14–27-fold at 50 ng/mL after 24 h, highlighting a direct paracrine link between angiogenesis and myeloma growth [63]. Large-scale genomic analyses have further revealed that at least 94.5% of patients with PCM harbor oncogenic or potentially oncogenic variants [64]. Similarly, in clinical trials, NGS-based molecular karyotyping demonstrated approximately 90% predictive accuracy compared with conventional cytogenetic and FISH analyses. Patients classified as high-risk by the genomic panel exhibited significantly shorter PFS (*p* = 0.0015) [65].

Cytokine profiling studies have further supported the use of IL-6 and VEGF as biomarkers of therapeutic response, showing significantly elevated levels in newly diagnosed and refractory patients compared with controls (*p*-value < 0.0001), and marked decreases in responders [42]. Collectively, these findings endorse a combined diagnostic model in which traditional biochemical methods (SPEP, IFE, sFLC) are complemented by advanced technological platforms (MS, NGS) to enhance diagnostic sensitivity and specificity. This integrative approach aims to maximize early detection, improve prognostic stratification, and ensure the economic feasibility of diagnostic procedures in the clinical management of PCM [66].

### 3.3. MS and Advanced Analytical Chemistry Methods

MS and other advanced analytical chemistry techniques have revolutionized the diagnosis and monitoring of PCM by enabling novel approaches to detect, quantify, and characterize M protein, sFLC, and other biomarkers involved in tumor progression. Unlike traditional immunological methods such as SPEP and IFE, MS can identify M protein at extremely low concentrations, with detection limits on the order of 0.0002 g/dL, allowing relapse to be detected 2–10 months earlier than with conventional techniques [67,68]. In addition to its high sensitivity, this method provides detailed information on structural variants of immunoglobulins and identifies post-translational modifications, such as glycosylation and phosphorylation, which are present in over 90% of the specimens analyzed. These modifications are highly significant, as they reflect the molecular heterogeneity of the tumor and may contribute to the variability in patient responses to targeted therapies [68,69].

Longitudinal biomarker monitoring (biomarkers are measured repeatedly over time) via MS enables real-time therapy modulation and has been shown to reduce the risk of progression by up to 82% in patients with undetectable MRD [70]. This dynamic approach is particularly useful for assessing therapeutic response and adapting treatments according to the biological trajectory of each patient. However, its practical application has limitations. The lack of standardized protocols and interlaboratory variability in protein quantification, estimated at around 20%, can affect the reproducibility and comparability of results across different centers [71]. Additionally, the need for costly equipment, specialized personnel, and established data analysis procedures limits its routine implementation in standard clinical laboratories [72]. Despite these constraints, MS remains one of the most powerful tools in current oncohematological diagnostics. Its integration with other branches of chemistry, including nanotechnology and biospectrometry, has enabled the development of hybrid systems capable of probing molecular interactions within the tumor microenvironment, achieving correlations of up to 75% with therapeutic response in pilot studies [72,73]. For example, NPs-assisted MALDI-TOF MS platforms have been developed to detect clonal immunoglobulin light chains and minimal residual disease in PCM, attaining sensitivities 10–100 times higher than conventional immunofixation and demonstrating strong correlations with clinical remission rates [74].

In addition, these technologies have markedly reduced the need for repeated bone marrow biopsies, by up to 100% in some studies [67], by providing a noninvasive means of assessing disease progression or remission. These clinical advantages, combined with the ability to integrate proteomic, genomic, and metabolomic data, position MS as a cornerstone of precision diagnostics and personalized medicine in PCM [67]. To conclude, MS, along with modern analytical chemistry methods, is indispensable for the precise determination and structural investigation of proteins related to the PCM. This high sensitivity and the ability to monitor MRD are the key aspects that make this methodology pivotal in current diagnostics. However, its broader clinical application will depend on the development of internationally standardized protocols, the training of specialized personnel, and wider accessibility of the technology. These steps are also essential for the full integration of these techniques into routine oncological practice.

### 3.4. Molecular Imaging Methods Applied to Diagnosis

Chemistry-based molecular imaging techniques represent a transformative advancement in science that has greatly improved the diagnosis and monitoring of PCM by enabling noninvasive detection of bone lesions, marrow infiltration, and tumor heterogeneity [75]. These techniques combine metabolic and molecular sensitivity with high anatomical resolution, allowing a comprehensive evaluation of disease status and therapeutic responses. The most commonly used modalities are single-photon emission computed tomography (SPECT/CT) and PET/CT [75,76]. SPECT/CT, typically performed using the radiotracer 99mTc-sestamibi (also known as technetium-99m methoxyisobutylisonitrile or MIBI), is a widely used radiopharmaceutical in nuclear medicine. It achieves a bone lesion detection rate of approximately 85% and offers the advantages of greater availability and lower cost compared to PET/CT [76]. In contrast, 18F-fluorodeoxyglucose (18F-FDG) PET/CT provides excellent metabolic sensitivity, with detection rates of 88–90% reported in clinical series [77]. It is capable of detecting early functional changes in the bone marrow before structural alterations become apparent, making it an indispensable tool for initial workup, relapse detection, and evaluation of therapeutic response. For example, FDG-PET/CT can reveal metabolically active focal lesions that remain undetectable by conventional MRI, enabling early identification of minimal residual disease and predicting relapse several months before clinical progression [77].

Innovations in medical imaging and nanotechnology have been driven by the development of functionalized NPs that specifically bind to surface antigens on plasma cells (e.g., CD38, CD138). These NPs have demonstrated the potential to enhance diagnostic specificity to 70–80% in preclinical studies by enabling targeted visualization of tumor cells while simultaneously delivering therapeutic agents to the same biological target. For example, gold and superparamagnetic iron oxide NPs conjugated with anti-CD138 antibodies have been employed for dual magnetic resonance and optical imaging of myeloma lesions, allowing for concurrent detection and doxorubicin delivery directly to malignant plasma cells. This approach has resulted in increased tumor suppression and reduced systemic toxicity in murine models [78,79]. This approach, known as theranostics (integrating diagnosis and therapy), represents one of the most promising advances in the molecular treatment of PCM. However, its clinical application remains largely trial-based and requires validation across multiple centers before it can be adopted as a standard procedure. Another emerging technique is Raman spectroscopy, a light-scattering optical method that exploits molecular interactions to provide information on the chemical composition and structure of tissues. This technique allows real-time analysis of the tumor microenvironment without the need for exogenous contrast agents, enabling the visualization of endogenous biochemical changes associated with disease progression [79,80]. For example, near-infrared (NIR) fluorescence imaging using indocyanine green–loaded NPs has been employed to monitor bone marrow angiogenesis and plasma cell infiltration in murine models of PCM, enabling dynamic assessment of vascular remodeling and early evaluation of treatment response. However, the spatial resolution and tissue penetration of NIR remain far inferior to those of PET or MRI, which currently limits its clinical applications [81]. MRI remains the gold standard for anatomical and structural imaging in PCM, with sensitivities of 80–85% for detecting bone lesions [82]. Although it does not provide immediate metabolic information, MRI is essential for monitoring disease progression and identifying bone or residual complications. Available data indicate that a synergistic approach combining conventional and novel imaging techniques enhances early diagnosis, risk assessment, and personalized therapy [82]. Even so, while SPECT/CT is a more cost-effective and widely accessible method, PET/CT provides more detailed metabolic information. Targeted NPs and Raman spectroscopy also offer a glimpse into the future of personalized, minimally invasive molecular diagnostics. The ultimate stepwise integration of these hybrid techniques, along with standardized protocols and validation in multicenter trials, will be essential not only for their introduction but also for confirming the benefits of multimodal imaging in PCM diagnosis and monitoring, thereby laying the foundation for patient-tailored precision medicine.

### 3.5. Comparison of Diagnostic Methods and Future Perspectives

The diagnosis of PCM requires an integrated approach that combines multiple techniques to detect both M protein and the molecular and structural changes associated with the disease. Classical biochemical methods, including SPEP, IFE, and sFLC analysis, remain essential for disease detection and monitoring, as they provide both quantitative and qualitative information on M proteins in serum and urine. These techniques allow for the assessment of tumor burden, disease progression, and therapeutic response, offering advantages such as relatively low cost, high reproducibility, and broad clinical availability. Moreover, their extensive historical use has generated large reference databases that facilitate standardization and interlaboratory comparisons, making them indispensable tools for both initial diagnosis and ongoing follow-up of PCM [83].

NGS and other molecular methods in combination with advances in MS and other emerging technologies have drastically improved the sensitivity and specificity of PCM diagnosis, leading to more accurate identification of MRD and genetic changes [84]. For example, deep sequencing panels, such as those used in the Myeloma Genome Project, can detect recurrent mutations in genes like KRAS, NRAS, BRAF, and TP53 at variant allele frequencies below 1%, while MS-based assays can identify residual monoclonal immunoglobulins at concentrations lower than 0.001 g/L, surpassing the sensitivity of conventional immunofixation and flow cytometry. These improvements enable physicians to follow clonal evolution throughout therapy and to adapt therapeutic approaches in an individual-patient-specific manner based on each patient’s own molecular profile [85]. Even so, biochemical methods such as SPEP, IFE, and sFLC assays are faster, less expensive, and more widely available; however, they have limitations in non-secretory PCM or when polyclonal immunoglobulins interfere with the analysis [86]. Molecular methods such as qPCR and NGS have much greater sensitivity, which allows residual clones to be detected at a level of 1 abnormal cell in 10^6^ normal cells, leading to enhancement of post-treatment surveillance and risk stratification [87].

On the other hand, imaging techniques, including PET/CT and MRI, provide both structural and functional information on bone infiltration, tumor metabolic activity, and overall disease extent, making them an essential complement to the comprehensive evaluation of PCM [82]. Such methods are also limited by the fact that biochemical and molecular techniques generally provide information on circulating biomarkers or genetic changes, but they cannot show the exact location or structural injury inflicted by the tumor. In contrast, MRI provides detailed visualization of bone marrow involvement and lytic lesions, while PET/CT identifies metabolically active disease sites that may not yet be visible structurally. The combination of these imaging techniques allows for a fuller and more accurate evaluation of disease burden, evolution, and response to therapy [82].

Table 2 provides a general overview of the major biochemical, molecular, and imaging-based detection techniques for PCM, including their technical parameters, sensitivities, specificities, detection limits, and clinical utility. A comparative analysis of these approaches illustrates the shift in PCM diagnostics from standard laboratory methods to advanced molecular analytical systems and functional imaging. This transition marks a move from a descriptive model toward a predictive and personalized approach, enabling earlier detection of MRD and more precise monitoring of therapeutic response [84,86].

Traditional biochemical methods such as SPEP, IFE, and sFLC quantification remain the cornerstone for the diagnosis of PCM. SPEP, also known as serum electrophoresis (SE), is relatively simple and inexpensive to perform. With a sensitivity of 70–80% and a specificity greater than 95%, it serves not only as a screening tool but also for monitoring purposes [86]. However, its detection limit of 0.2–0.5 g/L prevents identification of very low M protein levels, potentially leading to false-negative results in non-secretory or hyposecretory myelomas. For instance, in one study, approximately 5–10% of patients with confirmed PCM showed no detectable M-protein bands on SPEP despite having measurable disease by other methods, highlighting the limitations of this technique in low-secretion forms [86]. In this context, IFE provides a marked increase in sensitivity, up to 95%, enabling characterization of the immunoglobulin isotype and confirmation of M protein even at very low concentrations. However, its prognostic value is limited due to the lack of precise quantification and its restricted applicability for MRD detection. The main utility of IFE lies in early relapse detection and monitoring of patients with myeloma-associated nephropathy, although its interpretation may be affected by changes in renal function [58].

The introduction of MS has led to a qualitative leap in diagnostic sensitivity, enabling detection limits on the order of 10^−3^ g/L and establishing it as the most sensitive technique for quantifying monoclonal immunoglobulin isotypes and detecting MRD [67]. Its ability to discriminate multiple isotypes in a single run, combined with high reproducibility, has made MS a reference tool in research and high-complexity laboratories, although it is not yet routinely used in clinical practice due to high costs and technical demands [58,67]. Overall, conventional biochemical methods remain valuable for their accessibility, reproducibility, and speed, and are generally sufficient for detecting submicroscopic residual clones, although limitations exist in certain cases.

Imaging modalities such as PET/CT and MRI, based on anatomy and metabolism, provide additional information to biochemical and molecular techniques since they allow evaluation of both anatomical and metabolic aspects. Active bone lesions and post-treatment metabolic responses can be evaluated using PET/CT, which is particularly useful for distinguishing inactive lesions from viable residual disease, with a sensitivity of 85–90% [77]. On the other hand, without exposing the patients to ionizing radiation, MRI has superior sensitivity in detecting diffuse bone marrow infiltration as well as lesions < 3 mm. Although each technology has limitations in terms of cost and availability, the evidence suggests that they play a fundamental role in the comprehensive evaluation of PCM, particularly in patients who are molecularly negative yet exhibit clinical signs of disease progression [88]. NGS is the most molecularly informative technique for PCM. MRD detection with a sensitivity of up to 10^−6^ enables the simultaneous analysis of somatic mutations, gene rearrangements, and resistant subclones. This genomic resolution allows for precise molecular risk stratification, identification of patients harboring high-risk mutations (e.g., TP53, KRAS, or t(4;14)), and prediction of relapse before clinical manifestation [84]. Although relatively expensive and bioinformatically demanding, NGS is not yet routinely applied; however, its prognostic value makes it a standard tool in reference centers.

From a relative perspective, Table 2 illustrates that diagnostic sensitivity progressively increases from traditional biochemical methods to advanced molecular and imaging-based modalities. While SPEP and sFLC assays allow for rapid initial diagnosis, the incorporation of MS, NGF, and NGS enhances sensitivity to subclonal levels, thereby optimizing therapeutic monitoring and MRD detection. Sequential application of these tools, for example, SPEP + sFLC + NGF + PET/CT, can achieve diagnostic accuracy approaching 100%, minimizing false negatives and improving prognostic stratification [77,89]. This evolution illustrates that the diagnosis of PCM has transitioned from an exclusively clinical and radiological-based method to a multimodal, integrated, and predictive approach. Biochemical methods, including SPEP, IFE, and sFLC quantification, remain important for detecting and monitoring M proteins due to their speed and wide availability. However, their limited sensitivity necessitates integration with molecular and functional imaging tools such as MS, NGS, PET/CT, and MRI. Together, these strategies improve the accuracy of the diagnosis, facilitate relapse detection at an early stage, and allow for individual treatment options [84,90].

The rapidly advancing technology and the integration of biochemistry, genomics, and imaging are ushering in a new age for diagnosis. The integration of multiomic platforms with diagnostic nanotechnology tools (e.g., biosensors based on functionalized NPs) will enable multiplexed molecular detection and real time therapeutic monitoring. Together, these advances point toward a comprehensive, NGS approach focused on continuous PCM surveillance, reduced toxicity, and improved overall patient survival [91,92].

Overall, classical biochemistry techniques such as SPEP and IFE are still available, and they are cost-effective and timely for the initial diagnosis and routine monitoring, but they could not detect small amounts of protein and were incompatible with non-secretory myeloma and low-protein conditions, underlying that additional methods such as detection of free light chain in urine and serum could be necessary [58,86]. Advanced molecular techniques, including MS, NGF, and NGS, offer superior sensitivity and precision for MRD detection and clonal profiling, yet their higher costs, technical demands, and infrastructure requirements restrict widespread adoption [67,84,87,90]. Imaging techniques (e.g., PET/CT and MRI) offer essential structural and functional information but are limited by accessibility, duration of the examination, and, for PET/CT, exposure to radiation [77,88]. The optimal diagnostic strategy, therefore, lies in the integration of complementary techniques, balancing sensitivity, specificity, cost, and clinical applicability. Future research should focus on standardizing multi-modal diagnostic algorithms, validating cost-effective workflows for routine use, and exploring emerging technologies such as NP-based biosensors and multiomic platforms to enable real time, minimally invasive PCM surveillance with improved prognostic value [79,91,92]. In conclusion, the most effective diagnostic strategy in PCM requires the combination of biochemical, molecular, and imaging methods, balancing sensitivity, specificity, and cost. Future research should focus on standardizing multimodal algorithms and developing emerging technologies, such as NP-based biosensors and multiomics platforms, to enable continuous, precise, and minimally invasive disease monitoring [79,91,92].

## 4. Therapeutic Strategies in PCM

The management of PCM has evolved from nonspecific cytotoxic regimens to precision therapies guided by molecular and cellular insights into the disease. Current treatment strategies combine conventional chemotherapy with novel targeted and immunotherapeutic approaches, reflecting a clear shift toward personalized medicine [93,94]. Therapeutic strategies for PCM can be broadly classified into five main categories: (i) conventional chemotherapy and systemic therapies, (ii) radiotherapy (RT), (iii) immunotherapies using monoclonal antibodies and CAR-T cells, and (iv) nanotechnology-based and emerging molecular therapies. Each modality differs in mechanism, efficacy, and applicability depending on disease stage, yet all contribute to improving survival and quality of life for patients with PCM [94]. The following Section 4.1, Section 4.2 and Section 4.3 provide an overview of these therapeutic strategies, detailing their mechanisms of action, clinical effectiveness, limitations, and future perspectives within the framework of personalized medicine.

### 4.1. Chemotherapy and Conventional Pharmacologic Treatment

Chemotherapy continues to play a central role in the management of PCM, particularly for patients who are ineligible for novel agents or autologous stem cell transplantation. Over the past several decades, its development has evolved from the empirical use of alkylating agents to rationally designed combination regimens that employ drugs with complementary mechanisms of action to enhance efficacy while minimizing toxicity [93,94]. In the era of targeted and immunotherapeutic strategies made possible by biotechnological advancements, conventional cytotoxic drugs still play a crucial role in the induction, maintenance, and salvage treatment of PCM. The comparative analysis in Table 3 highlights the progressive specialization of chemotherapeutic agents used in PCM, reflecting a historical shift from nonspecific compounds to drugs with well-defined molecular targets. In addition, summarizes the pharmacological properties, mechanisms of action, pharmacokinetic parameters, and key adverse effects of the main chemotherapeutic agents employed in PCM treatment.

Alkylating agents, such as melphalan and cyclophosphamide, were the first compounds to demonstrate clinical activity against PCM [95]. Their mechanism involves DNA crosslinking, which inhibits replication and transcription, ultimately inducing apoptosis. Melphalan, a nitrogen mustard analog, remains a key component of pre-transplant conditioning regimens, achieving overall response rates (ORR) above 80%, although hematologic toxicity is common, with grade 3–4 neutropenia occurring in approximately 60% of patients and mucositis in about 30%. Cyclophosphamide, a prodrug activated by the cytochrome P450 system, has good oral bioavailability and a half-life of 3–12 h. It is frequently used in combination protocols, particularly with PIs, although 10–15% of patients may develop hemorrhagic cystitis [96].

The introduction of IMiDs marked a major advancement in chemotherapy. Thalidomide, the first agent in this class, combines antiangiogenic and immunomodulatory effects through the modulation of T and NK cells, achieving response rates between 30% and 50%. However, prolonged use results in irreversible peripheral neuropathy in over 60% of patients [97]. Lenalidomide, a second-generation analog, significantly improved both efficacy and safety, extending PFS to 26 months compared with 17 months for thalidomide [98]. Pomalidomide, effective in cases refractory to lenalidomide and bortezomib, achieves ORR of 30–35% with manageable myelotoxicity. Collectively, IMiDs demonstrate how structural optimization of drugs can enhance selectivity and immunomodulatory potency while minimizing adverse effects [99].

PIs represent another major pharmacologic milestone in PCM therapy. By blocking the 26S proteasome complex, which degrades misfolded proteins, they induce proteotoxic stress and apoptosis in malignant plasma cells. Bortezomib, the first PI approved, improved OS beyond 60 months and achieved complete responses (CR) in up to 38% of newly diagnosed patients, although it is associated with dose-dependent neuropathy in approximately 40% of cases [100]. Carfilzomib, an irreversible epoxyketone inhibitor, provides durable responses (ORR 56%) with lower neurotoxicity but carries an 8–10% risk of cardiotoxicity [101]. Ixazomib, the first oral PIs, improved PFS by 35% when combined with lenalidomide and dexamethasone, with mild thrombocytopenia and fatigue as the most common adverse effects [102].

Corticosteroids, such as dexamethasone and prednisone, complement chemotherapy by inducing apoptosis in plasma cells and reducing bone marrow inflammation. Dexamethasone enhances cytotoxic synergy with alkylating agents and IMiDs, increasing response rates by 20–25%, whereas prednisone offers a less potent but more tolerable option for maintenance therapy [103]. Broadly, rational combinations of alkylating agents, IMiDs, PIs, and corticosteroids achieve an ORR exceeding 70%, with PFS ranging from 18 to 26 months [98]. This balance of efficacy and tolerability illustrates how classical chemotherapy has evolved from empirical cytotoxic regimens to a scientifically informed, patient-specific approach. It remains the backbone of multimodal PCM therapy, providing the foundation upon which novel molecular and targeted strategies are built, ultimately improving both survival and quality of life [98,100].

### 4.2. RT Treatments

RT remains an important adjunctive therapy in PCM, primarily aimed at achieving local control of painful, compressive, or structurally unstable bone lesions rather than treating systemic disease. The radiosensitivity of malignant plasma cells allows moderate radiation doses to achieve high rates of local tumor control while minimizing damage to surrounding tissues [104,105]. The choice of optimal RT dose and fractionation should be tailored to the treatment intent, whether curative or palliative. In the largest international single-arm trial involving 84 patients with solitary plasmacytoma, Elsayad et al. (2020) reported local control in 96% of patients and complete remission in 46%, with doses above 40 Gy associated with significantly lower local relapse rates [105]. Similarly, Curry et al. (2020) demonstrated a 10-year local control rate of 88% in patients treated with definitive RT (median dose 45 Gy), supporting that moderate-dose RT provides long-term local disease control with acceptable late toxicity [106]. These results align with international RT guidelines, which recommend 40–50 Gy in 1.8–2.0 Gy fractions for curative treatment and 20–30 Gy in 10–15 fractions for palliative management of osteolytic lesions [104].

Recent clinical practice trends indicate a global shift toward shorter RT regimens for myeloma-related bone disease. In a population-based cohort of 623 Australian patients, reported that 47% of RT courses used 2–5 fractions, while single-fraction RT accounted for 18%, reflecting a move toward short-course schedules that maintain efficacy while enhancing patient convenience [107]. These patterns support the increasing role of RT for symptom relief in advanced disease, particularly when used in combination with systemic therapies [107]. RT provides rapid and durable palliation in the vast majority of treated lesions. Analyzed 577 irradiated lesions and reported pain control in 87% of patients at the end of treatment, increasing to 97% at three months, with a radiologic response rate of 79% [108]. The safety profile was favorable, with grade ≥ 2 adverse effects occurring in only 4.1% of sessions, even when RT was administered concurrently with systemic therapies. These results highlight that appropriately dosed RT is both safe and effective, even within multimodal treatment contexts [108].

Modern approaches advocate integrating RT into personalized therapeutic strategies. Highlight that advances in molecular characterization and imaging are enabling individualized RT planning, optimizing dose delivery based on the biological and genetic characteristics of the tumor [109]. This stringent organization enables RT to be locally effective with minimal systemic toxic effects [109,110]. Therefore, RT can be concluded to be an essential component of PCM therapy, providing effective local control, rapid symptom relief, and low toxicity when properly indicated. It is used as a second-line agent in systemic therapy and following high-dose therapy with autologous stem cell support to enhance functional results and quality of life. Advances in RT fractionation, integration with biologically targeted therapies, and personalized treatment planning are expected to further enhance its role in contemporary multimodal PCM management.

### 4.3. Immunotherapy

Immunotherapy has revolutionized the approach to treating PCM by using methods that stimulate the patient’s immune system and direct it towards the malignant plasma cells. Unlike traditional chemotherapy, which exerts nonspecific effects on rapidly dividing cells, immunotherapies aim to restore immune surveillance impaired by the tumor and eliminate residual clones that are resistant to cytotoxic agents or hematopoietic stem cell transplantation [111]. Immunotherapy in PCM encompasses mAbs, IMiDs, bispecific antibodies and next-generation cellular therapies, with diverse mechanisms of action and clinical uses. Bispecific antibodies are recombinant molecules that simultaneously bind a tumor-associated antigen on plasma cells, such as B-cell maturation antigen (BCMA) or G protein-coupled receptor class C group 5 member D (GPRC5D), and CD3 on T cells, thereby redirecting immune effector cells against the malignant clone [112].

Next-generation therapies, which refer to innovative treatments that move beyond traditional chemotherapy or first-generation immunotherapies, include bispecific antibodies (BiTEs, Bispecific T-cell Engagers), designed to simultaneously bind a cytotoxic T cell and a tumor plasma cell, facilitating targeted lysis without prior antigen recognition [113]. These next-generation agents are part of an advanced wave of precision immunotherapies that exploit engineered molecules or cells to enhance immune specificity and potency. Blinatumomab, targeting CD19 and CD3, serves as a prototype and has inspired the development of BiTEs more specific for PCM, such as those targeting BCMA, a marker unique to malignant plasma cells. Phase II clinical trials have reported ORRs near 65% in patients refractory to multiple prior lines—that is, patients who have not responded to several previous therapeutic regimens, including combinations of PIs, immunomodulators, and monoclonal antibodies, though with risks of cytokinerelease syndrome (CRS) and reversible neurotoxicity [114].

Another important class of immunotherapeutics includes anti-SLAMF7 antibodies, such as elotuzumab, which enhance natural killer (NK) cell activity by engaging the SLAMF7 receptor, thereby improving targeted plasma cell destruction. Although their efficacy as monotherapy is limited, combinations with lenalidomide and dexamethasone have demonstrated PFS improvements of up to 4.5 months compared with standard treatment [111]. In parallel, IMiDs, including thalidomide, lenalidomide, and pomalidomide, are also considered immunotherapeutic agents due to their capacity to stimulate T and NK lymphocytes, inhibit angiogenesis, and modulate cytokine secretion (e.g., IL-6 and TNF-α) that drives tumor proliferation [97,99]. These indirect immunomodulatory effects enhance the efficacy of mAbs and contribute to sustained disease control, particularly in post-transplant maintenance regimens [115]. On the other hand, CAR-T cell therapies represent a major breakthrough in immunotherapy for PCM. These treatments involve the genetic modification of autologous T lymphocytes to express chimeric antigen receptors (CARs) that target specific plasma cell antigens, such as BCMA, GPRC5D, or FcRH5 [111]. CAR-T products, including idecabtagene vicleucel and ciltacabtagene autoleucel (Carvykti^®^), have demonstrated CR in 30–40% of patients, with sustained remissions exceeding 12 months in highly refractory myeloma, thereby redefining treatment expectations [114,116]. However, these therapies carry significant risks, including cytokine release syndrome (CRS) in up to 90% of cases and neurotoxicity, and require substantial infrastructure and financial resources, which limits their broad availability [117].

In conclusion, immunotherapy has greatly broadened the available treatment options for PCM, offering the potential for deeper remissions, better control of MRD and meaningful improvements in OS. The combination of mAbs with immunomodulatory agents and corticosteroids, or their inclusion in pre-transplant induction protocols, has demonstrated considerable synergy, resulting in optimal therapeutic outcomes [115]. Although responses to CAR-T or bispecific antibody therapies were stunning, clinical obstacles, such as secondary immune resistance, partial antigen re-expression, immune-related adverse events (AEs) including CRS, or prolonged cytopenias, persist. To address these limitations, second-generation approaches are under development, including dual-target CAR-T cells (e.g., BCMA- and GPRC5D-directed constructs) designed to overcome antigen escape, as well as affinity-tuned bispecific antibodies optimized for both safety and efficacy [118].

Immunotherapy represents one of the greatest achievements in the therapeutic history of PCM. Its continued integration into established treatment algorithms, that is, structured clinical decision-making pathways guiding physicians on the optimal sequence and combination of therapies according to disease stage, patient profile, and response—coupled with the ongoing refinement of predictive biomarkers—marks a definitive shift toward personalized immunologic medicine. In this paradigm, myeloma control is assessed not only by survival but also by clonal eradication and functional immune recovery [113,114,115,116,117,118]. Moreover, the development of immunotherapies has catalyzed the emergence of novel nanotechnology platforms, which hold the promise of improved targeted and controlled delivery of immunotherapeutic agents, enhancing efficacy while minimizing adverse effects. The following section explores the novel concept of utilizing nanotechnology in PCM therapy, highlighting its role in controlled drug delivery systems and its potential impact on overcoming therapeutic resistance.

## 5. Nanotechnology and Emerging Therapies

**Figure 5:**
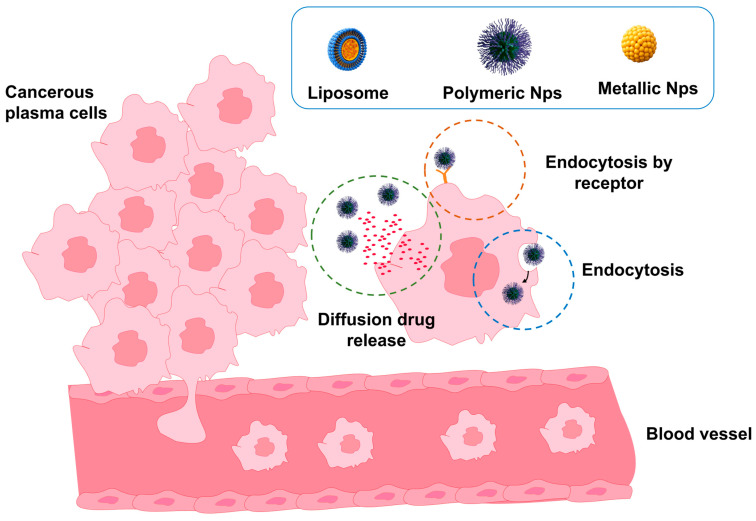
Schematic representation of nanomaterials used as controlled drug delivery systems in PCM therapy. NPs, liposomes, and nanocapsules encapsulate chemotherapeutic agents and selectively deliver them to malignant plasma cells, minimizing premature drug release and reducing systemic toxicity.

Nanotechnology has emerged as a promising strategy to enhance PCM therapy by enabling the development of controlled drug delivery systems that improve therapeutic efficacy, reduce systemic toxicity, and increase the bioavailability of antineoplastic agents [82,118]. These platforms are based on engineered nanomaterials smaller than 200 nm, which can selectively accumulate in tumor tissues through the enhanced permeability and retention (EPR) effect, a phenomenon in which the leaky vasculature and impaired lymphatic drainage characteristic of tumor microenvironments allow NPs to extravasate and remain localized at the tumor site. Aberrant tumor vasculature further facilitates this effect, providing a mechanism for passive drug targeting to the bone marrow, the primary sanctuary of malignant plasma cells [79]. Thus, the drug can be released in the cancer cell mainly through (i) nonspecific interactions (drug release outside the cancer cell), (ii) endocytosis, and (iii) receptor-mediated endocytosis, the latter involving a specific and directed interaction because the NPs are functionalized to bind to receptors on the cancer cell (Figure 5). For example, in a study using CD38- or CD138-targeted liposomal NPs in myeloma models, the targeted particles demonstrated significantly higher in vivo tumor accumulation and cellular uptake compared to non-targeted forms, thereby validating NPs delivery within the bone marrow microenvironment [79,108]. Within this context, the most widely employed nanotechnological systems, including liposomes, polymeric NPs, solid lipid nanocapsules, dendrimers, and functionalized MNPs, are presented and discussed with respect to their individual advantages in stability, biocompatibility, drug release kinetics, and loading capacity [82].

**Figure 6:**
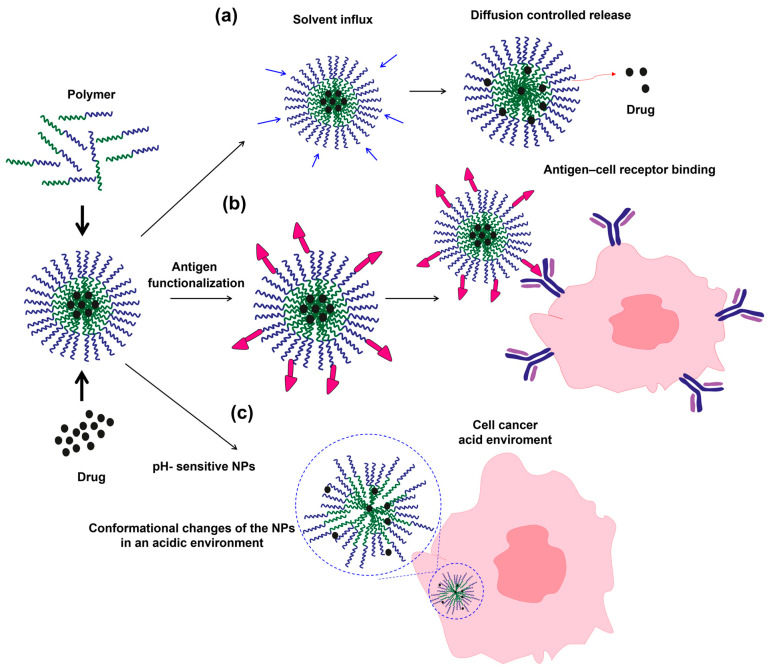
Formation and illustration of different drug-release mechanisms of liposomal and polymeric NPs: (**a**) diffusion-controlled release induced by solvent influx, (**b**) antigen functionalization of NPs, and (**c**) pH-responsive conformational changes in the acidic microenvironment of cancer cells.

Table 4 summarizes the main types of nanomaterials applied in PCM treatment, highlighting their drug release mechanisms, therapeutic efficacy, potential for toxicity reduction, and relevant references. Table 4 demonstrates that various nanocarrier systems, such as liposomes, polymeric NPs, and silica-based hybrids, enhance drug delivery efficiency and therapeutic outcomes in PCM compared to traditional formulations. Liposomes are extensively studied in PCM; these spherical vesicles consist of one or more phospholipid bilayers and can encapsulate hydrophilic molecules within the aqueous core or lipophilic molecules within the bilayer, thereby enabling sustained drug release and protection from enzymatic degradation [119]. These liposomal NPs have been applied to release drugs in the cancer microenvironment, where the drug is encapsulated during NP formation and released by solvent influx. In this process, the NPs swell in the medium, gradually expanding and allowing the drug to diffuse through the membrane (Figure 6a) [120]. However, several drugs cannot permeate cancer cells, reducing their therapeutic efficiency. Therefore, functionalizing NPs with proteins that specifically interact with receptors on cancer cells has become a promising alternative, as it increases NP permeation into the cells (Figure 6b). For instance, PSGL-1, targeted liposomes carrying bortezomib and the Rho-associated protein kinase (ROCK) inhibitor Y27632 achieved a 60% reduction in tumor proliferation in vitro, a 55% reduction in tumor size in vivo, and a 40% increase in survival. These formulations also exhibited lower toxicity to the bone marrow than free bortezomib, indicating enhanced selectivity and reduced systemic exposure [121].

On the other hand, beyond liposomal NPs, functionalization can also be applied to other types of nanoparticles; for example, folate-functionalized mesoporous silica NPs (~100 nm) also demonstrated promising results, achieving over 80% uptake in folate receptor-positive myeloma cells and a 70% reduction in cell viability. This was accompanied by a 65% increase in reactive oxygen species, indicating selective induction of apoptosis, while free bortezomib exhibited higher toxicity in normal cells [122]. Similarly, silica, collagen hybrid xerogels loaded with bortezomib induced 70% apoptosis in malignant cells and reduced local tumor growth by 60%, while simultaneously enhancing bone regeneration by 45% [125]. In polymer–lipid hybrid platforms, a library of 15 nanoparticle formulations enabled the identification of a lead NP (B1) capable of efficiently delivering siRNA to bone-marrow endothelial cells, achieving potent Cyclophilin A (CyPA) silencing in vivo and reducing PCM cell adhesion and invasion without inducing cytotoxicity in BMECs [123]. Similarly, polymer–lipid hybrid NPs encapsulating siRNA targeting Tie2 were used to validate the delivery platform, demonstrating effective knockdown of Tie2 expression 48 h after intravenous administration and confirming selective nanoparticle accumulation in the bone marrow through high-throughput DNA barcoding [123].

Additionally, beyond antigen functionalization, liposomes and polymeric nanoparticles can be functionalized with ionizable groups such as amino, sulfonate, carboxyl, and imidazolyl groups to form pH-sensitive NPs, enabling intelligent and controlled drug release [15]. This effect arises because cancer cells typically exhibit a low pH microenvironment, leading to protonation of these functional groups, which alters the hydrophilic–hydrophobic balance within the NPs, induces conformational changes, and promotes drug release into the cancer cell [120]. For instance, PEG-PLGA NPs functionalized with BCMA showed a high entrapment efficiency of 88.6% and pH-controlled release. In PBS (pH 7.4, similar to blood), they displayed a slow and sustained release profile. However, at pH 5 (similar to the acidic tumor microenvironment), a faster drug release was observed due to protonation of the PLGA carboxylic groups, which alters the NP structure [124]. Similar results were observed with doxorubicin loaded into polyethylene glycol-modified cadmium telluride quantum dots (PEG-CdTe QDs), a hybrid system in which the drug is entrapped within a polymeric matrix surrounding the quantum dots. Experimental results showed that the NPs released approximately 40% and 90% of the drug at neutral and acidic pH, respectively, indicating strong pH-responsive behavior [124].

On the other hand, MOFs have also been explored against PCM, and offer highly ordered porosity, tunable surface chemistry, and exceptional drug-loading capacity, enabling encapsulation of hydrophilic and hydrophobic agents with programmable release triggered by acidic pH or enzymatic cleavage, conditions characteristic of the cytokine-rich PCM marrow niche dominated by IL-6, VEGF, and pro-survival factors [40,41,63]. Their modularity allows conjugation with targeting ligands or bone-specific motifs, enhancing intracellular delivery while mitigating resistance linked to NF-κB, PI3K/Akt, and JAK/STAT activation [12,73,79]. ADCs build on the clinical success of antibody-based immunotherapies such as CD38- and SLAMF7-targeting antibodies, providing selective cytotoxic delivery to malignant plasma cells [111,112,113]. BCMA-directed ADCs remain the most advanced in PCM due to restricted normal tissue expression, consistent antigen density, and efficient internalization kinetics. Antigen shedding and intraclonal heterogeneity represent ongoing challenges, but the modular design of ADCs allows iterative optimization and combination with other immunotherapies to deepen responses [124].

Exosomes, by contrast, provide a biologically adaptive nanoplatform with inherent immune evasion, prolonged circulation, and efficient diffusion into dense marrow niches inaccessible to conventional NPs [40,75]. Engineered exosomes can display tumor-targeting ligands and carry therapeutic cargoes such as siRNA or proteasome inhibitors, simultaneously modulating gene expression and drug sensitivity while reaching “sanctuary zones” with limited vascular access [12,40,41,63]. Similarly, MNPs offer distinct advantages as synthetic delivery systems. MNPs, such as gold or iron oxide NPs, can combine drug delivery with imaging or photothermal therapy, enabling multifunctional and externally controllable treatment strategies [118]. In addition, they can be surface-functionalized for targeted delivery, exploit enhanced permeability in tumor vasculature, and complement other modalities like MOFs or exosomes in multimodal regimens. Collectively, these platforms—MOFs, ADCs, exosomes, liposomes, and MNPs—illustrate a convergent movement toward precision drug delivery that integrates controlled release, immune modulation, and microenvironmental targeting. Their successful implementation in PCM will depend on overcoming stability, tissue penetration, and manufacturing challenges while harnessing their complementary strengths to enable personalized, multimodal therapeutic regimens.

In terms of clinical advancement, liposomal systems have shown great progress. For instance, EPC/cholesterol/mPEG-DSPE liposomes encapsulating melphalan demonstrated pH-sensitive release, resulting in a 50% reduction in hepatic toxicity, a 2.5-fold increase in plasma half-life, and a 30% improvement in survival in animal models [119]. Similarly, optimized DSPC/cholesterol/mPEG-DSPE liposomes loaded with bortezomib increased plasma exposure 30-fold and achieved tumor inhibition of 37% and 57% in NCI-H929 and OPM-2 xenografts, respectively, compared to 17% and 11% with free drug [115]. Among the most innovative systems, platelet membrane-coated PLGA NPs (PM/BTZNP) enhanced tumor targeting and reduced immune recognition, achieving a 65% tumor reduction, 70% increase in apoptosis, and a 50% extension in survival in in vivo models [127]. Also, liposomal formulations carrying natural compounds, such as curcumin (Lipocur™), demonstrated 90% uptake in multiple myeloma cells versus only 10% in healthy lymphocytes, effectively inhibiting tumor proliferation while sparing normal tissues [126].

Advanced liposomal strategies have also enabled dual or synergistic therapies. Dual-drug liposomes containing carfilzomib and doxorubicin achieved 70% tumor inhibition and 50% lower systemic toxicity compared to free drugs [131], while bone-targeted liposomes co-delivering carfilzomib and BMS-202 enhanced T-cell infiltration by 40% and extended survival by 60% [129]. Additionally, vitamin E–based liposomes enriched with Pluronic F108 and co-loaded with melphalan and simvastatin reduced systemic toxicity by 40% and permitted repeated dosing through controlled release [129]. From a translational perspective, PEGylated liposomal dexamethasone (Dex-PL) tested in a phase I clinical trial in patients with multiple myeloma maintained therapeutic plasma levels for over seven days, produced clinical improvement in five of seven participants, and caused no severe adverse events [128]. Broadly, the data demonstrate that nanotechnology platforms significantly enhance therapeutic efficacy, with response rates of 65% to 85% and a 30–60% reduction in systemic toxicity compared to conventional chemotherapy. Liposomes are highly stable, exhibit prolonged circulation times, and preferentially accumulate in the bone marrow via the EPR effect, enabling sustained drug concentrations at the tumor site. Polymeric NPs and hybrid systems (e.g., lipo-polymer and polyplex platforms) provide additional advantages, including controlled release, surface functionalization with antibodies or ligands, and co-delivery of synergistic agents.

On the other hand, nanotechnology has demonstrated a decisive role in overcoming pharmacological resistance by enabling selective, sustained, and multicomponent delivery of therapeutic agents that avoid premature degradation, modify pharmacokinetics, and increase the effective drug concentration at the tumor site. The most relevant strategies include the use of multifunctional nanocarriers, co-encapsulation of synergistic drug combinations (such as bortezomib–lenalidomide or melphalan–dexamethasone), and stimulus-responsive targeted release triggered by changes in pH, temperature, or local magnetic fields [119,127]. Table 5 summarizes the main nanotechnology strategies developed to counter resistance to conventional PCM treatments, presenting their quantified experimental results in vitro and in vivo.

Dual-release NPs, by enabling the sequential administration of drugs with different mechanisms of action, generate therapeutic synergies that simultaneously disrupt pro-tumor signaling pathways and DNA repair mechanisms. In experimental models, these formulations achieved more than an 80% reduction in cell proliferation and up to 2.6-fold greater efficacy compared with conventional free-drug treatments [127]. On the other hand, pH-sensitive liposomes and dendrimers conjugated with P-gp inhibitors demonstrate that smart platforms can mitigate cellular resistance mechanisms, either through triggered release in the acidic tumor microenvironment or by directly inhibiting efflux pumps that reduce intracellular drug levels. This strategy enhances tumor-selective cytotoxicity and, consequently, minimizes systemic side effects and improves clinical tolerability [79,119]. Additionally, metallic NPs functionalized with mAbs targeting CD38 or BCMA represent a dual therapeutic and diagnostic (“theranostic”) approach, integrating drug-induced cytotoxicity with localized damage produced by controlled hyperthermia. In vivo, these systems reduced tumor burden by 75%, with no detectable relapse over 30 days, highlighting their clinical relevance in refractory cases [118]. Finally, hybrid polymer–lipid nanocapsules have shown great promise by co-encapsulating an immunomodulatory agent with a cytotoxic drug, achieving nearly 80% tumor inhibition and more than doubling survival in murine models of refractory disease [128].

### 5.1. Costs and Future Projections of Oncological Nanomedicines

The development of oncological nanomedicines and cellular therapies such as CAR T has transformed cancer treatment, offering significant clinical benefits, yet high costs remain a critical barrier to adoption. The global market for nanomedicine and NPs-based drug delivery systems is projected to grow from approximately US$ 105.95 billion in 2025 to US$ 209.73 billion in 2034, with a Compound Annual Growth Rate (CAGR) of 7.91% [132]. Other estimates suggest that the broader nanomedicine market could reach US$ 1022.5 billion by 2035, with a CAGR of ~12.75% [133], while NPs-based drug delivery alone could increase from US$ 109.14 billion in 2025 to US$ 178.32 billion in 2030, with a CAGR of ~10.32% [134,135]. This market growth indicates that unit costs of nanomedicines could decrease over time due to economies of scale, process optimization, and industrial automation. Companies producing liposomes or polymeric NPs could potentially reduce dose costs by 20–40% by 2030–2035 in an optimistic scenario of high demand and efficient production [135]. In more conservative scenarios with smaller-scale production and regulatory constraints, costs could remain between US$ 20,000 and 30,000 per treatment cycle consistent with cost-effectiveness analyses reported in the nanomedicine field [136].

The manufacturing costs of nanomedicines are high due to the need for specialized Good Manufacturing Practice-compliant facilities, strict particle-size and drug-loading control, sterility, and characterization, with initial capital investments in equipment such as microfluidic devices or homogenizers ranging from US$ 500,000 to 2 million [135]. These requirements explain why a treatment cycle with lipid-based or polymeric NPs can cost between US$ 25,000 and 30,000, significantly higher than conventional chemotherapy regimens (~US$ 3000). Cost analyses and translational evaluations support this difference in manufacturing complexity [135,136]. Advanced techniques such as magnetic nanoparticle hyperthermia (MHT), which integrate physical and pharmacological mechanisms, have also shown substantial associated costs [137]. By comparison, CAR-T therapies present even higher costs due to their personalized nature and clinical infrastructure requirements. For example, idecabtagene vicleucel (Abecma) is marketed at ~US$ 419,500 per infusion, and ciltacabtagene autoleucel (Carvykti) at ~US$ 465,000 per infusion [138,139]. Total costs including hospitalization, monitoring, and management of adverse effects can exceed US$ 500,000 per patient, as confirmed in clinical trials of anti-BCMA CAR-T therapies [139]. International scenarios show lower costs in countries such as India, where a local CAR-T (NexCAR19) has been estimated at US$ 40,000–50,000 [140], while in Spain a patient in the public system received treatment at ~€350,000 [141].

A potential advantage of nanomedicines is industrial scalability and standardization, which could allow wider accessibility in countries with less developed hospital infrastructure. Advances in nanoparticle platforms and scalable lipid-based delivery systems support this potential [142]. In terms of cost-effectiveness, while CAR-T provides a single-treatment option with potential for long-term remission, its high price and infrastructural demands limit global accessibility. Nanomedicines—though often requiring multiple doses—may be more feasible for middle- or low-income countries if production processes are optimized and market growth continues as projected [132,133].

Finally, future cost projections suggest that a nanotherapy cycle could cost US$ 15,000–20,000 in optimistic scenarios (efficient production and high demand) or US$ 20,000–30,000 in conservative scenarios (limited scale and regulatory constraints), representing significant potential reductions compared to current costs, yet still requiring health policies and efficient production chains to translate into real patient access [136,137]. In conclusion, both nanomedicines and CAR-T therapies represent significant therapeutic advances in oncology, but cost and accessibility remain critical determinants, dependent on clinical efficacy, production scalability, and health-system capacity.

### 5.2. Advantages, Limitations, and Future Perspectives of Nanotherapies in PCM

Nanotechnology-based therapeutic platforms have emerged as a promising strategy to improve treatment specificity and minimize systemic toxicity in PCM. Functionalized NPs, including gold, polymeric, and superparamagnetic iron oxide systems, offer key advantages over conventional chemotherapy. Their surface can be functionalized with targeting ligands such as anti-CD38 or anti-CD138 antibodies, enabling selective accumulation in malignant plasma cells within the bone marrow niche. For example, targeted PLGA-based NPs have shown 3–6-fold increases in intratumoral drug accumulation and 40–60% reductions in systemic exposure, compared with non-targeted counterparts [49,73,78]. This targeted delivery not only enhances drug concentration at disease sites but also reduces off-target exposure, lowering systemic toxicity and improving therapeutic indices. Moreover, nanocarrier-enabled controlled and sustained drug release maintains therapeutic concentrations for 48–72 h, improving cytotoxicity against myeloma cells and partially overcoming resistance mechanisms associated with high peak–low trough drug kinetics [79,104]. Additional benefits include the co-delivery of multiple agents, such as proteasome inhibitors with corticosteroids or IMiDs—which have demonstrated synergistic tumor reduction of up to 70–80% in xenograft models [11,49]—as well as the possibility of combining therapeutic and diagnostic (theranostic) capabilities.

In PCM theranostics, NP-based platforms modulate the molecular pathways that sustain plasma cell survival and therapeutic resistance. Several nanosystems described in this review, including liposomes, polymeric PLGA–PEG carriers, mesoporous silica NPs, and gold NPs, interfere with IL-6–driven NF-κB and JAK/STAT activation, two axes that maintain high MCL-1 and BCL-2 levels in malignant plasma cells [12,41,63,118]. For example, gold NPs functionalized with anti-CD38 antibodies achieve laser-triggered photothermal inhibition of tumor growth, producing a 75% tumor mass reduction and preventing recurrence for 30 days, partly by suppressing NF-κB–dependent survival signaling [118]. Likewise, polymer–lipid hybrid NPs delivering siRNA against CyPA significantly reduced tumor burden in vivo and, when combined with bortezomib, extended overall survival from ~65 days to ~90 days in a multiple myeloma mouse xenograft model, representing the strongest therapeutic effect among all treatment groups [123]. Liposomal and PLGA–PEG drug-loaded systems also indirectly inhibit PI3K/Akt by enhancing intracellular accumulation of bortezomib, resulting in 70–85% apoptosis induction and survival increases of 35–50% in xenograft models [120,121,124]. Through these combined effects pathway inhibition, gene silencing, and controlled intratumoral release theranostic NPs restore apoptosis despite high BCL-2/MCL-1 expression and overcome intrinsic and acquired drug resistance.

Despite these advantages, several limitations and challenges remain. Stability issues such as aggregation, opsonization, or premature clearance can compromise circulation time, with non-PEGylated platforms exhibiting up to 80% hepatic uptake within the first 2 h after systemic administration [88]. Manufacturing remains a barrier to clinical translation, as strict control of NPs size, surface chemistry, and charge is required for reproducible performance [78]. Current reports indicate batch-to-batch variability of 15–25% in ligand density and drug loading for antibody-functionalized NPs, complicating GMP scaling [88,107]. Limited penetration of certain nanomaterials into mineralized bone or deeply infiltrated marrow niches may reduce efficacy in skeletal regions heavily affected by PCM; for instance, studies show that bone penetrating NPs exhibit 30–50% lower delivery efficiency to osteolytic niches than to soft tissue tumor sites [80,101]. Furthermore, long-term biocompatibility, clearance mechanisms, and potential accumulation in non-target tissues require further investigation: iron oxide and polymeric NPs have displayed persistent splenic deposition for up to 30 days post-administration in rodent models [79,110]. Regulatory standardization and quality control particularly regarding nanomaterial characterization, immunogenicity, and long-term toxicity are also critical challenges for translation from bench to bedside.

Looking ahead, emerging strategies aim to overcome these limitations and expand the clinical potential of nanotherapies. Theranostic NPs that combine targeted drug delivery with real-time imaging offer the ability to monitor treatment response dynamically, with MRI-visible SPION platforms demonstrating 2–3× improved lesion detection and simultaneous therapeutic delivery [73,95]. Dual-targeting platforms, simultaneously recognizing plasma cell antigens (e.g., CD38, BCMA) and microenvironmental markers (e.g., CXCR4 or PDGFR-β), have shown over 90% internalization in myeloma cells and enhanced stromal penetration in preclinical models [73,100]. Biomimetic NPs, such as cell-membrane-coated systems, provide improved immune evasion and prolonged circulation, with reports of up to five-fold increases in half-life and enhanced marrow accumulation [73,104]. Hybrid nanocarriers functionalized with monoclonal antibodies further enhance selectivity and therapeutic depth [78,107]. Finally, the combination of nanotherapies with advanced immunotherapies including CAR-T cells and bispecific antibodies holds promise for synergistic approaches. Preclinical work shows that NPs mediated modulation of bone marrow cytokines (e.g., IL-6, CXCL12) improves CAR-T infiltration and cytotoxicity, resulting in 40–60% superior tumor clearance compared with CAR-T alone in animal models [82,121]. Such integration is particularly relevant considering that next-generation CAR-T and bispecific antibodies have achieved overall response rates exceeding 80–95% in heavily pre-treated PCM patients [121], setting a high benchmark that nanotherapies must meet or complement. In conclusion, while nanotherapies present substantial clinical potential in PCM, successful translation requires overcoming stability, biodistribution, and manufacturing challenges. Their future lies in multimodal, precisely targeted, and combinatorial approaches that leverage the complementary strengths of nanomaterials to enhance deep marrow penetration, improve tumor accumulation, reduce toxicity, and achieve personalized treatment strategies aligned with the efficacy standards set by emerging immunotherapies.

## 6. Conclusions and Prospects

PCM serves as a model hematologic malignancy in which genetic, epigenetic, biochemical, and microenvironmental changes interplay to dictate disease evolution and therapeutic response. Its molecular biology is now sufficiently understood to enable the identification of key biomarkers, such as β2-microglobulin, M protein, and free light chains, which are cornerstones for diagnosis, prognostic stratification, and therapy monitoring. These biomarkers, combined with advanced molecular technologies such as MS and NGS, have greatly enhanced diagnostic sensitivity and the detection of MRD, reaching sensitivities up to 10^−6^ and reducing false negatives by approximately 30–40% compared with traditional immunofixation methods. Consequently, diagnostic precision now exceeds 95%, guiding clinical management toward a more accurate and personalized paradigm.

In terms of treatment, the advent of PIs, IMiDs, and mAbs has significantly improved survival outcomes. Median OS has increased from 3–4 years in the early 2000s to over 8–10 years in patients treated with contemporary combination therapies. Nevertheless, clonal heterogeneity and drug resistance remain major barriers to cure, with recurrence rates as high as 60–70% within five years of first remission. In this context, nanotechnology represents a potentially disruptive strategy, improving drug delivery and selectively targeting malignant cells while reducing systemic toxicity by up to 40–50%. Nanomaterials such as liposomes, dendrimers, and hybrid NPs have demonstrated enhanced biodistribution and pharmacokinetics, achieving 2–3-fold higher drug accumulation at target sites and improved therapeutic efficacy in preclinical PCM models. The construction of nanoplatforms for theranostics, integrating both diagnosis and therapy within a single system, represents a promising step toward fully personalized medicine. When combined with emerging cellular therapies (e.g., CAR-T cells, which achieve response rates of 70–95%) and gene-editing technologies (e.g., CRISPR/Cas9, capable of precise targeting of oncogenic mutations with >90% editing efficiency in experimental settings), these advances envision a future in which PCM treatment focuses on the molecular eradication of malignant clones and prevention of relapse. Looking ahead, the integration of biochemistry and nanotechnology in PCM research not only deepens our understanding of disease pathophysiology but also drives the transition toward a biologically based, patient-centered therapeutic model. The next generation of research will be propelled by the multidisciplinary convergence of pharmaceutical chemistry, bioengineering, and translational biomedicine, aiming to develop smart, sustainable, and personalized therapeutic solutions. This emerging framework, founded on diagnostic precision and molecular insight, is transforming PCM therapy and paving the way for novel, efficacious, and individualized treatments.

## Figures and Tables

**Table 1:** Clinical, Biochemical, and Pathophysiological Biomarkers in PCM.

Biomarker/Subtype	Sample Type	Normal/Pathological Values	Method	Clinical Application	Prognosis	Clinical Manifestation	Ref.
M Protein (IgG/IgA)	Serum/Urine	Detectable: >0 g/L; MRD: 0.58 mg/L	SPEP, IFE, MS, MS/MS	Diagnosis, monitoring, MRD	Predicts relapse; correlates with tumor burden	Hyperviscosity, fatigue, renal damage	[1,6,35]
Serum LDH	Serum	Normal: 125–243 U/L; pathological ≥ 271 U/L	Enzymatic assay	Monitoring and prognosis	Elevated levels reflect high tumor proliferation	High tumor burden, aggressive disease	[1,36]
Osteocalcin	Serum	Normal: 11–43 ng/mL	Biochemical assay	Evaluation of bone metabolism	Decrease indicates osteoblastic inhibition	Bone pain, pathological fractures	[8]
Bone Alkaline Phosphatase/Tartrate-Resistant Acid Phosphatase	Serum	Normal: 40–129 U/L	Biochemical assay	Evaluation of bone resorption	Elevation reflects increased osteoclastic activity	Lytic lesions, bone pain	[8]
β2M	Serum	Normal: 1.0–2.5 mg/L; pathological > 5.5 mg/L	Biochemical assay	Risk stratification	High values associated with lower survival	Poor prognosis, renal failure	[22,31]
TP53/KRAS/MYC Mutations	Bone marrow/Serum	Presence of mutations	NGS	Risk stratification, prognosis	Associated with therapeutic resistance and high aggressiveness	High tumor burden, rapid progression	[23]
MicroRNA (miR-21, miR-15a/16)	Serum/Plasma	Normal vs. altered expression	qPCR	Prognosis and therapy resistance	Alterations associated with relapse and reduced apoptosis	Apoptosis resistance, recurrence	[25]
sFLC (κ/λ)	Serum/Urine	Normal ratio κ:λ 0.26–1.65; pathological <0.01 or >100	Freelite, N Latex, Sebia ELISA	Diagnosis, monitoring, risk stratification	Elevation indicates active myeloma; progression risk	Nephropathy, proteinuria	[29,30]
IL-6/VEGF	Serum/Plasma	Baseline levels variable; pathological ↑	qPCR, NGS	Indicators of tumor progression and angiogenesis	Elevation associated with early relapse	Osteoclastic activation, bone lesions	[34,36]
Serum Albumin	Serum	Normal: 3.5–5.0 g/dL; pathological < 2.8 g/dL	Biochemical assay	Prognostic stratification	Low values → lower survival	Fatigue, edema	[37]
24-h Proteinuria	Urine	Normal: <150 mg/day; pathological ≥ 500 mg/day	Proteinuria quantification	Prognosis	Elevated levels associated with lower survival	Nephropathy, proteinuria	[37]

IFE: Immunofixation Electrophoresis; IL-6: Interleukin-6; LDH: Lactate Dehydrogenase; PCM: Plasma Cell Myeloma; MRD: Minimal Residual Disease; MS/MS/MS: Mass Spectrometry/Tandem Mass Spectrometry; NGS: Next-Generation Sequencing; sFLC: Serum Free Light Chains/κ/λ ratio; SPEP: Serum Protein Electrophoresis; VEGF: Vascular Endothelial Growth Factor. In a complementary manner, the detection of TP53, KRAS, and MYC mutations by NGS or qPCR allows identification of patients with high risk of resistance to therapy and aggressive disease evolution [32]. The presence of these genetic alterations, together with abnormal β2M and LDH levels, defines poor-prognosis myeloma phenotypes. Laboratory techniques have demonstrated progressively lower detection limits, with sensitivities improving from 10^−3^ g/dL in SPEP to 10^−6^ g/dL in MS or next-generation flow cytometry [37]. This progress has resulted in the significantly enhanced sensitivity in detecting MRD, leading to the therapeutic intensity being modulated on the basis of biological and not merely clinical responses.

**Table 2:** Biochemical, Molecular, and Imaging Diagnostic Methods Used in PCM.

Type of Method	Sensitivity/Specificity	Detection Limit	Main Advantages	Disadvantages	^a^ Turnaround Time (h)	Clinical Application	Ref.
sFLC	95%/85–90%	1–5 mg/L	Detects occult disease and early relapse	Interlaboratory variability; renal impairment may alter values	24	Therapeutic monitoring, early relapse detection	[58]
MS	>99%/>99%	0.001 g/L	Detects multiple isotypes; precise quantification; high reproducibility	Expensive; requires specialized personnel	72–96	MRD detection and post-treatment monitoring	[67]
PET/CT	85–90%/90–95%	Lesions ≥ 4 mm	Evaluates disease extent and metabolic activity	Radiation exposure; limited availability	24–48	Assessment of bone lesions and metabolic response	[77]
SPEP	70–80%/>95%	0.2–0.5 g/L of M-protein	Cost-effective, widely available, useful for initial diagnosis	Does not detect low-level proteins or free light chains	24–48	Initial detection of monoclonal protein	[86]
IFE	90–95%/>98%	0.1 g/L	High sensitivity to confirm M-protein; identifies isotype	Limited for quantification; not useful for MRD	48–72	Confirmation of monoclonal immunoglobulin isotype	[86]
MRI	90–95%/90%	Lesions ≥ 3 mm	No radiation; detects diffuse infiltration and early lesions	Expensive; longer examination time	24–72	Evaluation of marrow infiltration and focal lesions	[88]
NGF	up to 10^−6^/>99%	1 abnormal cell per 10^6^	High sensitivity; rapid detection of residual clones	Requires fresh sample and standardization	24–48	MRD evaluation in bone marrow	[89]
NGS	10^−6^/>99%	1 abnormal cell per 10^6^	Detects somatic mutations and MRD; high prognostic value	High cost; limited infrastructure	120–168	Molecular risk stratification; MRD	[90]

^a^ Turnaround Time: time between taking the sample and issuing the diagnostic test result. SPEP: Serum Protein Electrophoresis; IFE: Immunofixation Electrophoresis; sFLC: Serum Free Light Chains; MS: Mass Spectrometry; NGF: Next-Generation Flow Cytometry; NGS: Next-Generation Sequencing; PET/CT: Positron Emission Tomography–Computed Tomography; MRI: Magnetic Resonance Imaging.

**Table 3:** Main Drugs Used in PCM Treatment.

Drug	Class	Mechanism of Action	Main Uses	Pharmacokinetics	Major Toxicity	Common Adverse Effects	Common Combinations	Ref.
Melphalan	Alkylating Agents	DNA alkylation, cross-linking that blocks replication and transcription	Conditioning for autologous transplant; first-line therapy	Variable oral bioavailability (25–89%); renal excretion	Cumulative myelosuppression	Mucositis (30%), neutropenia (60%), thrombocytopenia (45%)	Melphalan + Thalidomide/Lenalidomide/Prednisone	[95]
Cyclophosphamide	Alkylating Agents	DNA alkylation after hepatic activation	Salvage therapy or in transplant-ineligible patients	Good oral absorption; hepatic activation (CYP450); half-life 3–12 h	Hemorrhagic cystitis (10–15%)	Nausea, alopecia, neutropenia, bladder toxicity	Cyclophosphamide + Bortezomib + Dexamethasone	[96]
Thalidomide	IMiDs	Immunomodulation, angiogenesis inhibition, apoptosis	Elderly or non-transplantable patients	Slow absorption; t^1/2^ 6–7 h; hepatic and renal metabolism	Irreversible peripheral neuropathy (>60%), severe teratogenicity	Sedation, constipation, fatigue	Thalidomide + Dexamethasone/Melphalan	[97]
Lenalidomide	IMiDs	Stimulates T and NK cells, inhibits angiogenesis and tumor support	First-line, post-transplant maintenance	Good oral bioavailability; renal excretion; t^1/2^ 3–5 h	Deep vein thrombosis (8–12%), myelotoxicity	Neutropenia (35%), thrombocytopenia (25%), diarrhea	Lenalidomide + Bortezomib + Dexamethasone	[98]
Pomalidomide	IMiDs	Potent immunomodulator and cytotoxic	Lenalidomide- and bortezomib-refractory myeloma	Good oral absorption; t^1/2^ 7.5 h; renal and fecal elimination	Moderate myelotoxicity	Anemia (40%), neutropenia (30%), fatigue	Pomalidomide + Dexamethasone	[99]
Bortezomib	PIs	Inhibits 26S proteasome → accumulation of misfolded proteins → apoptosis	Refractory or newly diagnosed	IV or SC; hepatic metabolism; t^1/2^ ≈ 40 h	Peripheral neuropathy (40%)	Fatigue, diarrhea, thrombocytopenia	Bortezomib + Lenalidomide + Dexamethasone	[100]
Carfilzomib	PIs	Selective and irreversible proteasome inhibition	Refractory or relapsed	IV; hepatic metabolism (non-CYP); t^1/2^ ≈ 1 h	Cardiotoxicity (8–10%)	Dyspnea, fever, hypertension	Carfilzomib + Lenalidomide + Dexamethasone	[101]
Ixazomib	PIs	Oral proteasome inhibitor	Combination therapies in PCM	Oral; good bioavailability; t^1/2^ ≈ 9.5 days	Thrombocytopenia (25%)	Fatigue, mild neuropathy, diarrhea	Ixazomib + Lenalidomide + Dexamethasone	[102]
Dexamethasone	Corticosteroids	Induces apoptosis in malignant plasma cells; anti-inflammatory	Adjuvant in most regimens	High oral bioavailability; t^1/2^ 36–54 h	Hyperglycemia, prolonged immunosuppression	Fluid retention, insomnia, proximal myopathy	Present in almost all standard regimens	[103]
Prednisone	Corticosteroids	Similar to dexamethasone, lower potency	Combined with alkylating agents or IMiDs	Oral; hepatic metabolism; t^1/2^ 2–4 h	Hyperglycemia, immunosuppression	Fluid retention, hypertension	Prednisone + Melphalan/Thalidomide	[103]

DNA: Deoxyribonucleic acid; CYP450: Cytochrome P450; IMiDs: Immunomodulatory Drugs; IV: Intravenous; PCM: Plasma Cell Myeloma; NK: Natural Killer; SC: Subcutaneous; t^1/2^: Half-life.

**Table 4:** Nanotechnology in the Treatment of PCM.

Type of NPs	Nanomaterial Used	Active Compound	Experimental Model	Main Outcomes (Quantitative Results)	Ref.
Liposomal (Optimized)	DSPC/cholesterol/mPEG-DSPE liposomes	Bortezomib	In vivo: NCI-H929 and OPM-2 xenografts	Plasma exposure 30× higher than free BTZ. Tumor inhibition: 37% (NCI-H929) and 57% (OPM-2) vs. 17% and 11% for free BTZ.	[115]
Liposomal (pH-responsive)	EPC/cholesterol/mPEG-DSPE liposomes (2:5:2)	Melphalan	In vitro: ARD, PBMC; In vivo: BALB/c mice (ARD-luc xenografts)	hepatic toxicity reduced by 50%, plasma half-life increased 2.5×, survival improved by 30%.	[119]
Liposomal (Targeted)	PSGL-1-targeted liposomes (DPPC, DSPE-mPEG2000, DSPE-PEG-succinyl, cholesterol)	Bortezomib + ROCK inhibitor (Y27632)	In vitro: MM.1S, H929, OPM-2, HUVEC; In vivo: NCG mice (MM.1S xenografts)	Tumor proliferation reduced by 60%. In vivo tumor mass decreased by 55% and survival increased by 40%. Lower bone marrow toxicity vs. free BTZ.	[121]
Silica-based (Functionalized)	Folate-functionalized mesoporous silica NPs (~100 nm)	Bortezomib	In vitro: RPMI-8226, U266B1 (FR+), HeLa	Over 80% uptake in FR+ cells. Viability reduced by 70%, ROS increased by 65% vs. control. Free BTZ less selective and more toxic to normal cells.	[122]
Polymer–lipid hybrid	Polymer–lipid NPs (low MW PEI + C15 epoxides + PEG-lipids)	siRNA targeting Cyclophilin A and Tie2	In vitro: BMEC-60, MM.1S; In vivo: C57BL/6 mice (MM.1S xenografts)	siRNA encapsulation >90%. Gene silencing of 75%. Tumor burden decreased by 50%, survival prolonged by 35%.	[123]
Polymeric (Antibody-functionalized)	PLGA–PEG NPs functionalized with anti-BCMA antibodies (~168 nm, −13 mV)	Bortezomib	In vitro: MM.1S, CD138+; In vivo: NSG mice (MM xenografts)	Sustained release for 72 h. 85% internalization in BCMA+ cells. Apoptosis increased by 70%, survival extended by 45%.	[124]
Silica–collagen hybrid	Silica–collagen xerogels (sicXer) and bortezomib-loaded xerogels (boXer)	Bortezomib	In vitro: 10 MM lines, 8 patient samples; In vivo: 5T33 mouse model	Apoptosis induced in 70% of PCM. Local tumor growth reduced by 60%, bone regeneration increased by 45%.	[125]
Liposomal (Natural compound)	Lipocur™ liposomes	Curcumin	In vitro: RPMI-8226, NCI-H929, B lymphocytes	90% uptake in PCM vs. 10% in lymphocytes. Inhibited proliferation without toxicity to healthy cells.	[126]
Biomimetic (Platelet membrane-coated)	PLGA NPs coated with platelet membranes (PM/BTZNP)	Bortezomib	In vitro: LP-1, RAW264.7; In vivo: NOD/SCID mice (LP-1 xenografts)	Tumor reduction by 65%, apoptosis +70%, survival +50%. Enhanced targeting and low systemic toxicity.	[127]
Liposomal (Clinical trial)	Long-circulating PEGylated liposomes (Dex-PL)	Dexamethasone	Phase I clinical trial, 7 MM patients	Plasma levels maintained >7 days, no severe adverse effects. Clinical improvement in 5 of 7 patients.	[128]
Liposomal	Vitamin E oil liposomes modified with Pluronic F108	Melphalan + Simvastatin	In vitro and in vivo (acute toxicity)	Improved drug loading and sustained release. Systemic toxicity reduced by 40%. Enabled repeated simvastatin dosing.	[129]
Liposomal (Bone-targeted)	c(RGDfk)- and PEG-modified liposomes	Carfilzomib + BMS-202	In vitro: macrophages; In vivo: C57BL/6J and C57BL/KaLwRij mice	Bone marrow targeting. Tumor reduction 55%, T-cell infiltration +40%, survival +60%.	[130]
Liposomal (Dual-drug)	Dual-drug liposomes (DSPC, mPEG2000-DSPE, DPPE-GA)	Carfilzomib + Doxorubicin	In vitro: MM.1S, NCI-H929; In vivo: SCID CB-17 mice	Synergistic 1:1 ratio. Tumor inhibition 70%, systemic toxicity 50% lower vs. free drugs.	[131]

ARD: Human Multiple Myeloma Cell Line ARD; BCMA: B-cell Maturation Antigen; BTZ: Bortezomib; FR: Folate Receptor; HeLa: Human Cervical Carcinoma Cell Line; HUVEC: Human Umbilical Vein Endothelial Cells; PCM: plasma cell myeloma; NPs: Nanoparticle; OPM-2: Human Multiple Myeloma Cell Line OPM-2; PBMC: Peripheral Blood Mononuclear Cells; RAW264.7: Murine Macrophage Cell Line; ROS: Reactive Oxygen Species; RPMI-8226: Human Multiple Myeloma Cell Line RPMI-8226; U266B1: Human Multiple Myeloma Cell Line U266B1.

**Table 5:** Nanotechnology Strategies Against Pharmacological Resistance.

Nanotechnology Strategy	Type of Nanosystem	Main Mechanism of Action	Quantitative Results	Model/Application	Ref.
Efflux Pump Inhibition	PAMAM dendrimers conjugated with P-gp inhibitors	Blockade of active drug transport, increasing intracellular concentration of bortezomib	3.2-fold increase in intracellular concentration and restoration of sensitivity in 70% of resistant cell lines	Resistant cell models	[79]
Targeted Photothermal Therapy	Gold Nps (AuNPs) functionalized with anti-CD38 antibodies	Controlled release and localized destruction induced by laser irradiation	75% reduction in tumor mass and no recurrence for 30 days	Animal models of myeloma	[118]
pH-controlled Release	pH-sensitive liposomes loaded with melphalan	Controlled release in acidic tumor microenvironments (approx. pH 6.5), enhancing tumor selectivity	60% reduction in acquired resistance and 45% decrease in systemic toxicity	Animal models of PCM	[119]
Synergistic Co-encapsulation	PLGA–PEG Nps loaded with bortezomib and lenalidomide	Sequential and synergistic dual release of both agents, increasing bioavailability and intratumoral retention	80–85% reduction in cell proliferation and 2.6-fold increase in efficacy compared to free drugs	In vitro and in vivo studies in murine models	[127]
Combined Hybrid Nanocapsules	Polymer–lipid nanocapsules loaded with dexamethasone and carfilzomib	Immunomodulatory and cytotoxic synergy, with prolonged and controlled release	78% tumor inhibition and 2.1-fold increase in median survival	Refractory murine models	[128]

CD38: Cluster of Differentiation 38; P-gp: P-glycoprotein.

## Data Availability

Not applicable. No new data were created or analyzed in this study. Data sharing is not applicable to this article.
